# Functional Evaluation of Proteins in Watery and Gel Saliva of Aphids

**DOI:** 10.3389/fpls.2016.01840

**Published:** 2016-12-15

**Authors:** Aart J. E. van Bel, Torsten Will

**Affiliations:** ^1^Institute of General Botany, Justus-Liebig-UniversityGiessen, Germany; ^2^Institute of Phytopathology, Justus-Liebig-UniversityGiessen, Germany; ^3^Institute for Resistance Research and Stress Tolerance, Federal Research Centre for Cultivated Plants, Julius-Kühn InstituteQuedlinburg, Germany

**Keywords:** aphids, gel saliva, hemipterans, proteome, salivary proteins, watery saliva

## Abstract

Gel and watery saliva are regarded as key players in aphid–pIant interactions. The salivary composition seems to be influenced by the variable environment encountered by the stylet tip. Milieu sensing has been postulated to provide information needed for proper stylet navigation and for the required switches between gel and watery saliva secretion during stylet progress. Both the chemical and physical factors involved in sensing of the stylet’s environment are discussed. To investigate the salivary proteome, proteins were collected from dissected gland extracts or artificial diets in a range of studies. We discuss the advantages and disadvantages of either collection method. Several proteins were identified by functional assays or by use of proteomic tools, while most of their functions still remain unknown. These studies disclosed the presence of at least two proteins carrying numerous sulfhydryl groups that may act as the structural backbone of the salivary sheath. Furthermore, cell-wall degrading proteins such a pectinases, pectin methylesterases, polygalacturonases, and cellulases as well as diverse Ca^2+^-binding proteins (e.g., regucalcin, ARMET proteins) were detected. Suppression of the plant defense may be a common goal of salivary proteins. Salivary proteases are likely involved in the breakdown of sieve-element proteins to invalidate plant defense or to increase the availability of organic N compounds. Salivary polyphenoloxidases, peroxidases and oxidoreductases were suggested to detoxify, e.g., plant phenols. During the last years, an increasing number of salivary proteins have been categorized under the term ‘effector’. Effectors may act in the suppression (C002 or MIF cytokine) or the induction (e.g., Mp10 or Mp 42) of plant defense, respectively. A remarkable component of watery saliva seems the protein GroEL that originates from *Buchnera aphidicola*, the obligate symbiont of aphids and probably reflects an excretory product that induces plant defense responses. Furthermore, chitin fragments in the saliva may trigger defense reactions (e.g., callose deposition). The functions of identified proteins and protein classes are discussed with regard to physical and chemical characteristics of apoplasmic and symplasmic plant compartments.

## Introduction

Evidence is accumulating that host-plant proteins and salivary proteins of aphids play a major role in the “battle” between them. Aphid saliva contains proteins aimed to pave the way for the aphid stylet and to undermine plant defense and resistance (Tjallingii, 2006). Conversely, a high number of plant proteins are encountered along the stylet pathway. A part of the plant proteins is associated with defense responses, while others are involved in, e.g., metabolic and regulatory processes. Although some cortex-expressed proteins are able to deter or combat aphids, the majority of proteins of high relevance for plant defense against aphids may occur in the sieve-tube sap in view of the much longer stylet residence times there (Tjallingii, 2006; Will et al., 2013). Up to thousands of proteins have been detected in sieve-tube exudates of, e.g., grasses (Aki et al., 2008), *Arabidopsis thaliana* (Batailler et al., 2012) and cucurbits (Lin et al., 2009; Dinant and Lucas, 2013). Unproven as yet, some of them may function as deterrents, while several others are involved in immediate plant defense on the protein level (Will et al., 2013). Again other proteins with a high impact on aphid–plant interaction may be integral part of local and long-distance signaling pathways/cascades (e.g., van Bel et al., 2011, 2014).

Harmel et al. (2008) detected more than 200 different polypeptides in the saliva of the green peach aphid *Myzus persicae*, of which only nine proteins were identified having a known function in other insects (*Aedes aegyptii* and *Aphis mellifera*). Others were related to expressed sequence tags (EST) of *A. pisum* and *M. persicae*. Later, Carolan et al. (2011) identified 925 proteins by mass spectrometry in salivary gland extracts of the pea aphid *A. pisum*. Over 300 proteins, most of them with an unknown function, were reported to possess secretory signals. The latter property was regarded as an essential characteristic of proteins belonging to the salivary gland secretome (Carolan et al., 2011). Recently, Atamian et al. (2013) studied the protein composition of salivary gland extracts of the potato aphid *Macrosiphum euphorbiae*. They allocated 125 of the 460 detected proteins to the secretome due to the presence of a signal peptide in accordance with the previous definition (Carolan et al., 2011).

However, the numbers of salivary proteins should be treated with care, because proteins without a secretory sequence may also be part of the salivary secretome (Chaudhary et al., 2015). Secretory or signal peptides located at the N-terminus of proteins mediate their transfer to specific regions inside the cell or their secretion out of the cell. Signal peptide sequences are predicted by software tools such as SignalP, which selected proteins in salivary glands having an N-terminal signal peptide (used by Carolan et al., 2011; Atamian et al., 2013), while TargetP is employed for prediction of the subcellular location of eukaryotic proteins. In eukaryotes, proteins with or without a secretory peptide end can be secreted in an unconventional way (Nickel and Rabouille, 2009), which might explain the presence of proteins without a secretory signal in aphid saliva (Chaudhary et al., 2015).

Like in related hemipteran families (Sogawa, 1965; Ramm et al., 2015), aphids produce salivary proteins in two pairs of glands at either side of the aphid head (Ponsen, 1972). Secretory proteins are released into the salivary channel and then secreted via their mouth parts into the plant tissues. The larger (principal) and smaller (accessory) pair of glands probably produce two mixtures of saliva. Gel saliva is predominantly secreted into the apoplasm during stylet movement along the cell walls. Watery saliva is mainly secreted intracellularly during penetration of different plant cell types (Tjallingii, 2006), but is also reported to be secreted into the apoplasm (Moreno et al., 2011). The principal glands may mainly produce gel saliva as indicated by immuno-histochemical labeling of a 154 kDa protein, while watery saliva is hypothesized to be produced for the major part by the smaller accessory glands (Cherqui and Tjallingii, 2000). As for the protein production, a specialization seems to exist between the cells of the principal glands (Pan et al., 2015). While some proteins like C002 (Mutti et al., 2008) or ACYP100646 (Pan et al., 2015) are only produced in the principal glands, other proteins like ACYPI39568 are expressed in both primary and accessory glands (Guo et al., 2014). The secretory activity of the glands is possibly regulated by the environmental conditions around the stylet tip (Will et al., 2012), perceived by receptors located in the precibarium (Wensler and Filshie, 1969).

On its way to the sieve tube, the stylet follows an apoplasmic path (Tjallingii, 2006; Hewer et al., 2011). In the apoplasmic space or cell wall, continuously secreted drops of gel saliva are pierced successively by the stylet to form a tubular corridor after hardening (Will et al., 2012) that envelops, protects and guides the stylet. Cells along the pathway are regularly probed by the stylet (Tjallingii and Hogen Esch, 1993). During stylet penetration of, e.g., mesophyll cells, watery saliva is secreted into the intracellular space, followed by ingestion of some cell sap (Tjallingii, 2006). There are indications that aphids navigate to the sieve tubes following a “cell rejection mode” (Hewer et al., 2011). As long as the cells do not contain a threshold level of sucrose and do not have a pH of approximately 7.5 (the alkaline conditions in the sieve-tube sap; Hafke et al., 2005), the aphids “reject” such cells after cell-sap probing. After penetration of vascular cells having the pH-value and sucrose concentration incentive to feeding, the stylet progress halts (Hewer et al., 2010, 2011). It was speculated that the directional orientation of the stylet is enabled by an inborne autopilot, guiding the stylet in radial direction (Hewer et al., 2011).

Once the stylet has reached the sieve tubes, watery saliva is secreted for a period of 40–60 s (Tjallingii and Hogen Esch, 1993). Aphids then start ingestion of sieve-tube sap that is interrupted by regular intervals of saliva secretion (Tjallingii, 2006). Saliva is not delivered into the plant any more but mixes with ingested sieve-tube sap in the common duct at the tip of the stylet (Tjallingii, 2006). Given the specificity of aphid-plant interactions – many aphids are monophagous, while some are poly- or oligophagous – several intriguing questions regarding the protein composition of aphid saliva emerge. Does protein composition of watery saliva differ between aphid species? Can the protein composition of watery saliva be adapted when general feeders like *M. persicae* switch the host plant species? Is it possible to separate gel and watery saliva and, if so, is there a clear distinction in protein composition between the two saliva types? And as the most prominent question here, which are the functions of salivary proteins identified thus far and how do they interfere with plant actions? These and associated questions are addressed here.

## Secretion of Gel and Watery Saliva

### Sensing the Stylet Environment for Stylet Navigation

Observations by Hewer et al. (2010, 2011) and Will et al. (2012) point to the pH and the carbohydrate species as cues for stylet navigation to the sieve tubes and secretion of a saliva mixture that is adapted to the needs for stylet progress, orientation or feeding in dependence of the location of the stylet tip. Moreover, aphids appear to be able to perceive the presence of amino acids in artificial diets (Cherqui and Tjallingii, 2000). Because the stylet tip exclusively contains mechanoreceptors (Powell et al., 2006), aphids are likely capable of sensing the chemical stylet environment by chemosensillae in the precibarium (Wensler and Filshie, 1969; Backus and McLean, 1985), which requires frequent ingestion of cellular probes.

Together with other prameters, pH sensing would enable aphids to assess the stylet location. Through the clear distinction between cytosolic (pH 7.5; e.g., Felle and Bertl, 1986; Plieth et al., 1997; Bethmann et al., 1998; Felle, 2001; Hafke et al., 2001) and vacuolar pH (pH 5.5; e.g., Foyer et al., 1982; Nishimura, 1982; Weigel and Weis, 1984; Guern et al., 1986; Mathieu et al., 1989), aphids are able to identify the cell type punctured. Given the mechanical forces required to drive the stylet through the cell wall, it is expected that the stylet tip will instantly cross the thin cytosolic layer of parenchyma cells during penetration (Petterson et al., 2007) and reach the vacuole, which makes up almost the entire cell volume. Upon sensing the acidic vacuolar pH, aphids will retract the stylet and continue their search for a source of nutrition, until a sieve tube (pH 7.5, Hafke et al., 2005) is identified (for a simple model, see Hewer et al., 2011). The standard pH of 7.3 to 7.5 in sieve elements (Hafke et al., 2005) is due to the lack of vacuoles.

One of the crucial, albeit disputable, claims in this “rejection” hypothesis is that the stylet becomes inserted into the vacuole of parenchyma cells. It has been argued that aphid-transmitted viruses must be released from the stylet into the cytoplasm of parenchyma cells (Martin et al., 1997; Marchetti et al., 2009) to enable virus multiplication (Martelli and Castellano, 1971; Shalla et al., 1980) and the stylet tip must therefore reside in the cytoplasm during the entire cell-probing period (Powell et al., 2006). This view excludes stylet piercing of the tonoplast. However, pictures of stylet tips inside the vacuole (Hewer et al., 2011), traces of irreversible damage of intracellular membranes (Spiller et al., 1985; Hewer et al., 2011), and the presence of viruses inside the vacuole (Wan et al., 2015) corroborate the view that the vacuolar pH is sensed by aphids and thus seem to support the “rejection” hypothesis.

As a second cue for stylet orientation, the sucrose concentration has been proposed (Hewer et al., 2010, 2011). Aphids are able to discriminate between sugar species and sugar quantities (Mittler and Dadd, 1964; Auclair, 1969; Hewer et al., 2010). Many aphids show a clear preference for sucrose at high concentrations (Hewer et al., 2010), which is the common transport sugar in the majority of higher plants (Zimmermann and Ziegler, 1975) and mostly occurs in high concentrations in sieve-tube sap [e.g., barley 1080 mM (Lohaus et al., 1995), plantain 645 mM, celery 389 mM (Nadwodnik and Lohaus, 2008), and spinach 830 mM (Gerhardt et al., 1987; Lohaus et al., 1995)]. In contrast, vacuoles of parenchymatous cells contain concentrations of sucrose varying between 0.9 mM in plantain, 45 mM in celery, 64 mM in peach (Nadwodnik and Lohaus, 2008), and 40 mM in spinach (Gerhardt et al., 1987). Together with the low pH, a low sucrose concentration may be an incentive to retract the stylet and move on to the next cell. Like pH values, sucrose concentrations may be monitored by chemosensory cells in the precibarium (cf. Backus and McLean, 1985).

It has become obvious from tests with artificial diets that amino acids are indispensable substances for aphid feeding (Turner, 1971; Wille and Hartmann, 2008; Will, 2016a). The question arises, whether specific amino acids – as sucrose presumably does – act simultaneously as nutrients and as indicators for stylet orientation. The latter issue has not been investigated yet to the best of our knowledge.

In addition to chemical cues such as pH, sucrose and – perhaps – amino acids, the fourth sensory element in orientation may be turgor pressure, as aphids are able to perceive changes in turgor pressure in sieve tubes as demonstrated by an artificial feeding system (Will et al., 2008), and thus seem to sense turgor differences between the successive cells along the stylet pathway. All in all, the attractiveness of the “rejection” concept is that it provides a universal model for aphid orientation to the sieve tubes that is not dependent on species-specific traits.

### Sensing the Stylet Environment for Appropriate Saliva Secretion

When aphids are feeding from an artificial diet, a salivary sheath is formed that is attached to a Parafilm cover at the side facing the diet (e.g., Cherqui and Tjallingii, 2000; Cooper et al., 2010). The pearl necklace structure of gel saliva secreted *in vitro* suggests rhythmic pulses of saliva secretion (McLean and Kinsey, 1965; Will et al., 2012; Morgan et al., 2013). According to a long-lasting concept (McLean and Kinsey, 1965), the salivary sheath was formed, because gel drops were inflated from the inside by watery saliva, quickly solidified and then were pierced by the stylet to be followed by the next drop of gel saliva (McLean and Kinsey, 1965; Miles, 1972). Inflation, however, would lead to round cavities that do not form a tight-fit tube around the stylet. The discrete and straight tubular structure, visible by confocal laser scanning microscopy (Will et al., 2012), infers that the gel saliva hardens after that the stylet has pierced the gel saliva at the front side before release of the next drop without intervention of watery saliva. Occasional side branches of the gel-saliva puffs *in vitro* (Will et al., 2012) and regular side-branches of gel saliva tracts in plant tissues (Hewer et al., 2011) are indicative of an auto-programmed process switching between stylet movement, gel saliva secretion, and regular cell probing along the stylet pathway (Tjallingii, 2006).

The question arises, whether the predicted chemical cues (pH, carbohydrate species) are not only responsible for stylet navigation, but also control the secretion of different types of saliva (gel saliva in the apoplasmic, watery saliva in the symplasmic compartments). Saliva was collected in artificial diets mimicking apoplasmic and symplasmic solutions. Gel saliva depositions and diet fluids were collected at pH 5 (mimicking apoplasmic pH conditions) and pH 7 (mimicking neutral to weakly alkaline sieve-tube conditions). To study the protein composition of salivary sheaths a protocol was developed by Will et al. (2012). After solubilisation of salivary sheaths attached to Parafilm by breaking up the structural framework of the sheaths in several steps, the free proteins were separated by 1D SDS-PAGE (Will et al., 2012). At pH 5, hardly any proteins occurred in the diet fluid, whereas the Parafilm-adhesive gel saliva showed a multitude of protein bands in 1D SDS-PAGE gels (Will et al., 2012). By contrast, the diet fluid contained a rich diversity of protein bands at pH 7, whereas the number of proteins increased in gel saliva depositions (Will et al., 2012). pH 5 mimics the acidic conditions inside the apoplasmic compartment (Cosgrove, 2005), where secretion of gel saliva is needed to facilitate stylet progress (Miles, 1999). Furthermore, low pH may be optimal for the activity of enzymes in gel saliva as it is for other enzymes being active in the cell-wall compartment such as cell-wall invertase (Hothorn et al., 2010). In conclusion, the stylet-tip milieu elicits the secretion of watery saliva only under diet conditions that mimic the composition of sieve-tube sap.

### Separation of Watery and Gel Saliva Proteins

The capacity of aphids to discriminate between the diets paves the way for an almost unequivocal separation and assessment of gel and watery saliva proteins (Will et al., 2012). The risk of cross-contamination (watery saliva proteins enclosed in the sheath structure and proteins leaking from the sheath structure leaking into the diet) is high under sieve-tube conditions in the diet. Under conditions mimicking the apoplasmic environment, the amount of proteins in the diet fluid (containing watery-saliva proteins) was so low, that the gel saliva depositions are virtually devoid of watery-saliva proteins and, hence, the protein bands obtained from sheath solubilisation primarily represent gel-saliva proteins. Thus, the composition of watery saliva may be disclosed by subtraction procedures facilitated by previous identification of gel-saliva proteins. However, it should be stressed that some proteins may be part of both types of saliva as discussed above.

### A Side-Step: Physical Components of Aphid–Plant Interaction and Putative Consequences for the Salivary Proteome

Little attention has been paid to the physical components of interaction between aphids and plants (Will and van Bel, 2006) and the inherent consequences for protein composition of the saliva types. The stylet penetration site is usually located at the wall junction of two epidermal cells and the labium is anchored to the plant surface by a salivary flange made of gel saliva (e.g., Will et al., 2013). The wall junction probably offers the weakest spot in the cell-wall barrier and provides a strategic spatial location for stylet penetration. The stylet mainly moves along the primary cell wall (Tjallingii and Hogen Esch, 1993) that has a jelly and loosely woven structure. It represents the softest part of the cell walls with the lowest mechanical resistance. It is possible that stylet movement leverages the rigid cell-wall sandwich bordering either side of the primary wall. Because the stylet proceeds along the cell walls, aphids must generate a considerable force to thrust the stylet forward through the tortuous path inside the walls, where the fragile mouth parts could be damaged. Cell-wall degrading enzymes could be useful to reduce the wall resistance, but this view is under dispute (Cherqui and Tjallingii, 2000).

To create the strength, needed for stylet propulsion, the movement of the four subunits of the stylet (two maxillary and two mandibulary mouth parts) must be well coordinated. The maxillary and mandibulary pair at one side move together forward alternating with the other pair. Stabilization and support of the mouth parts during movement must be an important function of the salivary sheath. In conclusion, stylet movement requires a mixture of proteins that softens and digests the walls, lubricates the pathway to decrease the mechanical resistance, and forms a corridor through which the fragile stylet finds its way along the brisk cell wall angles and that stabilizes the movement of the mouthparts.

That the sheath does not possess the necklace structure *in planta* as obtained *in vitro*, where shots of gel saliva assume a spherical shape, may be due to physical constraints imposed by the cell walls around the sheath. Hardening of the salivary sheath is ascribed to oxidation of sulfhydryl groups (Miles, 1965; Tjallingii, 2006). Under ambient *in vitro* oxygen concentrations (∼20%; Hewer et al., 2011), “the pearls of the necklace” are discretely roundish having a rimmed texture (Will et al., 2012). Under reduced oxygen conditions, in contrast, the spheres were less delimited and the texture smoother, while the single drops are eliminated in the presence of DTT that impedes cross-bridging of sulfhydryl groups (Will et al., 2012). Due to the low oxygen level in the plant cortex (∼7%; van Dongen et al., 2003), sheath polymerization is anticipated to be less rapid than *in vitro* and initial bulging of the gel saliva drops will vanish during hardening. Moreover, the turgescence of the plant cells enveloping the sheath may compress the gel saliva during solidification. Due to retarded hardening under low-oxygen conditions, the sheath takes the shape of the mold presented by the cell walls.

Turgor of plant cells may also provide auxiliary information on cell identity. High sugar concentrations and high turgor are mostly linked, so that sieve elements stand out by a high turgor pressure. Moreover, pressure sensing might be relevant for initiating the secretion of saliva. Recognition of the atmospheric pressure or even negative pressure inside the apoplasm may trigger the secretion of gel saliva. Alternatively, secretion of gel saliva may result from sensing mechanical resistance as experienced during piercing of the Parafilm cover on diet solutions (Will et al., 2007; Carolan et al., 2009; Cooper et al., 2010; Chaudhary et al., 2015). The latter event (Cherqui and Tjallingii, 2000; Cooper et al., 2010; Will et al., 2012; Morgan et al., 2013) may mimic the resistance experienced during cell-wall penetration.

Cell punctures along the stylet pathway (Tjallingii, 2006) may cause local waves of electrical depolarization, which propagate to the nearby sieve tubes (van Bel et al., 2014) and prepare them to aphid attack. Plasmodesmata would provide a symplasmic lateral pathway (Kempers et al., 1998) for propagation of the depolarization wave, in which voltage-activated Ca^2+^-permeable channels are involved (van Bel and Ehlers, 2005; van Bel et al., 2014). Cell punctures made by stylets may be quickly repaired by plasma membranes in analogy to their reaction toward microelectrode impalement. Immediately after insertion of microelectrodes into cells or vacuoles, the pierced membranes seal off around the electrode tip and completely shut off, shortly after the electrode is retracted (e.g., Bates et al., 1983). Since stylet tips and conventional microelectrode tips have approx. the same diameter (1 micron, Will and van Bel, 2006), plasma-membrane wounds inflicted by punctures will probably close shortly after stylet retraction. According to this concept, secretion of gel saliva to seal off the puncture (Tjallingii, 2006) is therefore needed to prevent bulging of the turgescent protoplast associated with undesired physiological consequences rather than sealing the protoplast. Moreover, the chemical incompatibility of the hydrophilic gel-saliva and the lipophilic membrane material renders their fusion to seal the plasma membrane highly unlikely (Will and van Bel, 2006).

Stylet puncture in sieve elements has a number of physical consequences. First of all, free Ca^2+^ ions present in the cell walls will readily invade the sieve-element lumen via the puncture (Will and van Bel, 2006). Furthermore, the inevitable turgor drop linked with stylet penetration will activate mechano-sensitive Ca^2+^-permeable channels (Demidchik and Maathuis, 2007) causing Ca^2+^ influx into the sieve element (Furch et al., 2009; van Bel et al., 2014). In plant/aphid systems, where Ca^2+^ levels are instantly reduced by Ca^2+^ scavenging salivary proteins (Will and van Bel, 2006), the consequences of stylet penetration could be limited. In plant/aphid interactions with low Ca^2+^-binding capacities, however, we speculate that the temporary Ca^2+^ upsurge in sieve elements is sufficient to activate voltage-activated Ca^2+^-permeable channels (McAinsh and Pittman, 2009). The subsequent cascade of ion movements would be responsible for a strong local depolarization and the initiation of an electrical potential wave along successive sieve elements (e.g., van Bel et al., 2014, and references therein; Hedrich et al., 2016). Consequently, stylet impalement into a sieve element may trigger the propagation of an electric potential wave resulting in a series of reactions in cells adjacent to the sieve tube (van Bel et al., 2014). All above types of Ca^2+^ influx will collectively boost Ca^2+^ concentration in sieve-element mictoplasm (Furch et al., 2009; Hafke et al., 2009), which will in turn evoke gating of Ca^2+^-activated Ca^2+^ channels (CICR channels; Muir and Sanders, 1996) giving rise to massive Ca^2+^ release from the sieve-element endoplasmic reticulum. In conclusion, diverse ways of Ca^2+^ influx, brought about by physical events owing to stylet penetration, potentiate the elevation of Ca^2+^ level in sieve elements leading to cascades of local and remote events. Given the involvement of enhanced Ca^2+^ in a range of plant defense responses (Will and van Bel, 2006; van Bel et al., 2014) Ca^2+^-binding proteins in aphid saliva would be helpful to suppress plant defense.

## Species-Specificity of Salivary Proteins and Potential Adaptation to Host Plants

### Protein Profiles of Watery Saliva of Various Aphid Species Reared on One Host Plant Species

To our knowledge, the very first comparative study that included more than one aphid species feeding the same host plant species was conducted by Madhusudhan and Miles (1998). They detected differences and overlaps between the salivary protein bands in SDS-PAGE profiles of the pea aphid (*A. pisum*) and the spotted alfalfa aphid (*Therioaphis trifolii maculata*), both feeding on *Medicago sativa*. This observation coincided with distinct differences and overlaps (e.g., pectin methylesterase and endopolygalacturonase) in enzyme activities between saliva probes from the two species. Similarly, the SDS-PAGE band patterns of salivary proteins from *A. pisum*, *M. viciae*, and *A. fabae*, all reared on *Vicia faba*, disclosed a wide diversity between species-specific protein profiles (Will et al., 2009). Experiments similar to those of Will et al. (2009) using *A. pisum*, *M. viciae*, and *M. persicae* reared on *V. faba* produced almost identical, but less discrete protein bands (Vandermoten et al., 2014). Although protein identification was not executed, some proteins seem to be identical based on their molecular weight, while others appear to differ between aphid species.

A more sophisticated approach using mass spectrometry for protein identification (Rao et al., 2013) demonstrated that the salivary proteomes of *A. pisum* and the cereal aphid species *Sitobion avenae* and *Metopolophium dirhodum* showed several overlaps [e.g., trehalase and GMC (glucose-methanol-choline) oxidoreductase]. The results indicate that some elements of the salivary proteome have universal functions, while others may be adapted to the host-plant species. It raises the question if aphids are able to adapt their salivary composition to the host-plant species.

### Are Salivary Proteins Involved in the Adaptation to Host-Plant Species?

Biotypes of *A. pisum* have been reported to be adapted to diverse legume species due to minor variations in the genetic background of aphids (Via, 1991). Biotype variation may be regarded as long-term adaptation to different host-plant species, which was demonstrated by a genomic approach including different biotypes of the polyphagous aphid species *A. pisum* (Peccoud et al., 2009). They identified 11 different biotypes feeding on different legume species, showing significant adaptations of the aphid genome. In a genome-wide study on pea aphid, Jaquiéry et al. (2012) identified regions enclosing salivary protein genes and olfactory receptor genes that are likely involved in host-plant adaptation. The authors point out that the genetic markers used only cover a small percentage of the aphid genome (Jaquiéry et al., 2012).

Adaptation to host plants is of paramount importance in host alternation, an obligatory seasonal shifting between aphid species and host plants of distant genetic relationship. The gene expression patterns of the mealy aphid *Hyalopteris personikus* feeding on *Phragmites australis* in summer and several members of the Rosaceae in winter showed enormous seasonal variations (Cui et al., 2016). In summer, several secretory proteins, attributed to watery saliva, were highly expressed, while a salivary sheath protein was highly expressed in winter. All in all, aphids seem to be able to adapt their salivary proteome to the host plant.

## Remarks Concerning Collection and Separation Procedures of Salivary Proteins

### Proteins Obtained from Extracts of Salivary Glands or by Collection of Secreted Saliva: Advantages and Disadvantages

Protein composition of aphid saliva has been assessed by analysis of either the proteome of salivary gland extracts or stylet exudates. Analysis of the salivary proteome of gland extracts has the presumptive advantage that the samples are hardly prone to oxidation, degradation by proteases, or bacterial breakdown, all risks when using artificial diets. Furthermore, Carolan et al. (2011) identified a higher number of proteins in salivary gland extracts than in the proteome collected with artificial diets. Salivary glands extracts may be better suited to detect low-abundance proteins, as extracts often have higher protein concentrations and do not need to be concentrated prior to analysis by SDS-PAGE or MS/MS. In addition, extracts enable the detection of peptides of molecular weights lower than 3–10 kDa. These are the molecular cut-off sizes for ultrafiltration, commonly used for concentrating saliva-diet mixtures (Will et al., 2007; Carolan et al., 2009; Cooper et al., 2011; Chaudhary et al., 2015).

For the above reasons, the number of proteins in gland extracts is anticipated to be higher than in diet fluids. This difference may also illustrate a severe drawback of the method: proteins identified in gland extracts may profoundly deviate from the actual and functional salivary proteome. As an additional disadvantage of extract sampling, excision of intact salivary glands is demanding and one needs at least 300 glands to enable proteomic analysis (Rao et al., 2013).

An unmistakable advantage of saliva collection by using artificial diets is the unequivocal identification of proteins engaged in plant-aphid interaction. A further advantage of using artificial diets is that diets can be manipulated to provide preferential conditions for the secretion of either gel or watery saliva (Will et al., 2012). As noted above, however, proteins secreted in artificial diets may be subject to degradation due to long incubation times of 16–48 h needed for saliva collection (e.g., Will et al., 2007; Harmel et al., 2008; Carolan et al., 2009; Chaudhary et al., 2015). Furthermore, the procedures are laborious: pooled diets containing saliva of 1000s of aphids have to be concentrated by ultrafiltration to reach quantities needed for reliable protein analysis (Will et al., 2007; Carolan et al., 2009; Chaudhary et al., 2015). A major drawback of using diets may be the presence of traces of host-plant sieve-element proteins in the collected saliva (Chaudhary et al., 2015). As a final important issue, the composition of the saliva secreted into diet fluids in the absence of various host-plant cues may deviate from that secreted into plants (Guo et al., 2014; Wang et al., 2015b). Surprisingly, it has been demonstrated (Morgan et al., 2013) that gel saliva can also be collected *in aera*, thus without the intervention of artificial diets. This opens perspectives for a more amenable mode of collection and separation of gel saliva.

### Salivary Proteomics and Functional Assessment of Salivary Proteins

As soon as aphids invade a plant, a cross-fire of attacks and counterattacks bursts out. In the present paper, only the “weapons” from the aphid-side are discussed. While the number of salivary proteins that has been identified continuously increases, the most exhaustive list of hemipteran salivary proteins thus far (Sharma et al., 2014) includes more than 60 salivary proteins identified in diverse hemipterans. We will focus here on the few classes of aphid salivary proteins, of which the functional relevance has been identified.

One should bear in mind that almost none of the proteomic studies aimed or succeeded thus far to discriminate between gel and watery saliva. Almost all diet studies thus far were executed using diets at pH 7, a value at which gel and watery saliva are mixed (Will et al., 2012). Nevertheless, separation of the two types seems to be an absolute prerequisite for meaningful functional assessment, the more as the saliva types may have overlapping protein compositions as a further issue of complication (Will et al., 2012). Separately collected extracts of primary and accessory glands may give a rough impression of the degree of distinction between gel and watery saliva proteins. In contrast to diet studies, extract contents do not guarantee the presence of these proteins in the saliva as argued above. Hence, the following discussions suffer from uncertainties regarding the origin of the salivary proteins and the location of secretion inside the plant. Attribution to either gel or watery saliva follows an apparent logic rather than one based on solid experimental evidence.

## Functional Aspects of Salivary Proteins

### Predicted Gel Saliva Components

The major component of the salivary sheath is most likely a 154-kDa protein (ACYP1009881-PA; Carolan et al., 2009, 2011) having a high content of cysteine residues. This protein, termed “sheath protein”, SHP (Carolan et al., 2011), was first detected in the saliva of *A. pisum* (Carolan et al., 2009), and later in the saliva of *S. avenae* and *M. dirhodum* (Rao et al., 2013). Oxidation of the SHP sulfhydryl groups would lead to the formation of disulfide bridges (Miles, 1965; Tjallingii, 2006), catalyzed by disulfide isomerases, several of which were found in the salivary gland secretome of *A. pisum* (Carolan et al., 2011). Linked SHPs form the backbone of the salivary sheath. This scenario was supported by a diminished degree of polymerization, exemplified by a disorganized sheath structure, in the presence of the reducing compound dithiothreitol that breaks disulphide bonds (Will et al., 2012).

Inhibition of sheath formation by silencing of *shp* expression in *A. pisum* by means of injection of double-stranded RNA demonstrated that SHP is an important structural sheath protein (Will and Vilcinskas, 2015). Reduced sheath hardening weakened the capability of aphids to withdraw nutrition from sieve elements (Will and Vilcinskas, 2015). *S. avenae* individuals feeding on barley plants containing a similar dsRNA sequence suffered from decreased reproduction and reduced survival rates in comparison to aphids on control plants (Abdellatef et al., 2015). A necessity of salivary sheath proteins for the interaction between sucking insects and plants was also demonstrated for the brown plant hopper on rice plants (Huang et al., 2015).

Recently a second *A. pisum* cysteine-rich protein carrying 14 cysteine domains has been discovered (Guo et al., 2014). This zinc-dependent protein (ACYP139568), found enriched in extracts of the salivary glands of *A. pisum*, may constitute another part of the sheath backbone. The expression was enhanced when the aphids were feeding on plants instead of on artificial diets (Guo et al., 2014), which is a phenomenon; reported more often in transcriptome studies (Wang et al., 2015b).

It is to be expected that gel saliva contains wall-softening and -degrading enzymes facilitate stylet progress (see section: A side step: physical components of aphid–plant interaction and putative consequences for the salivary proteome). Therefore, it is no surprise that several cell-wall degrading enzymes were found among salivary proteins, although only a few have been attributed to gel saliva with certainty. Among the potential cell-wall degrading enzymes are pectinases (Ma et al., 1990; Cherqui and Tjallingii, 2000), pectin methylesterases (Ma et al., 1990; Madhusudhan and Miles, 1998), and polygalacturonases (Ma et al., 1990; Madhusudhan and Miles, 1998). Cellulase-(like) activity was detected in aphid saliva (Adams and Drew, 1965) and in aphid homogenates (Campbell and Dreyer, 1985). Several cellulase transcripts were recently detected in *M. persicae* and *Myzus cerasi*, but were absent in *R. padi* (Thorpe et al., 2016) which raises questions regarding their function.

Thus far, none of the effects of cell-wall degrading enzymes has been demonstrated unequivocally. Miles (1999) had even doubts on the effectiveness of the wall-degrading enzymes in general, given the presumptively low rates of catalytic activity and the rapid stylet progress so that the residence times of the stylet tip are short. In this context, it is worthwhile to note that cell-wall degradation products (e.g., cellodextrins and polygalacturonides) act as pathogen-induced molecular patterns evoking plant defense responses (Aziz et al., 2007; Will and van Bel, 2008). Polygalacturonides elicit an increase in cytosolic Ca^2+^ (e.g., Messiaen et al., 1993) which might act as a second messenger in plant defense (Maffei et al., 2007; War et al., 2012).

As a last note, cell walls contain a wealth of phenolic substances (Nicholson and Hammerschmidt, 1992), several of which interfere with aphid infestation by mechanical hindrance, while others are aphid deterrents. It is anticipated that gel saliva contains detoxifying proteins such as phenoloxidases to combat deterring and poisonous phenolics in cell walls and protoplasmic compartments (Miles and Oertli, 1993). Further information on saliva-mediated detoxification of phenolics is given in the Section “Detoxifying Proteins.”

### Predicted Watery Saliva Proteins

#### Ca^2+^-Binding Proteins

The targets of aphids are the sieve elements, nutrient-rich cells in plants. As explained above, puncturing the sieve tubes implies that the sieve elements are damaged by sudden impalement of the stylet. Damage of sieve elements provokes a dual sieve-plate occlusion mechanism in dicotyledons that can extend over considerable distances from the site of wounding inside a plant (Furch et al., 2007, 2010; van Bel et al., 2014). An immediate sealing by protein plugs as a first response is followed by a slower deposition of callose along the sieve pores (Furch et al., 2007, 2008, 2009). Both reactions are Ca^2+^-dependent and respond to an increase of Ca^2+^ in the sieve-element lumen (Knoblauch et al., 2001, 2003, 2012; Furch et al., 2007, 2009; Hafke et al., 2009). Ca^2+^ increase may arise from influx of cell-wall Ca^2+^ via the imperfectly sealed wound inflicted by the stylet (Will and van Bel, 2006) or via Ca^2+^ channels in the sieve-element plasma membrane or in the sieve-element ER-membranes (Buchen et al., 1983; Sjolund and Shih, 1983; Furch et al., 2009; Hafke et al., 2009). Ca^2+^ influx further away from the site of wounding is triggered by electro-potential waves propagating along the sieve elements (Hafke et al., 2009; van Bel et al., 2014). Sieve-element depolarisations spread into adjacent cells (Rhodes et al., 1996; van Bel et al., 2014), which might explain the occlusion of neighboring sieve tubes during feeding of the generalist aphid species *Macrosiphon euphorbiae* and *M. persicae* (Medina-Ortega and Walker, 2015).

Sieve-tube occlusion by protein plugs was observed in legume sieve tubes that contain forisomes, giant spindle-like protein complexes (e.g., Lawton, 1978; van Bel and van Rijen, 1994; Knoblauch and van Bel, 1998). Forisomes, which seem to be composed of distinctly demarcated subunits (Schwan et al., 2009; Müller et al., 2014), expand in response to wounding and fully occlude the sieve tubes (Knoblauch et al., 2001, 2012). Their expansion is reversible (Knoblauch et al., 2001): forisomes re-condense after some time (30 min to a few hours) provided that the sieve elements are not severely damaged (Furch et al., 2007, 2008, 2009). Forisome expansion is induced by Ca^2+^ influx into the sieve elements (Knoblauch et al., 2001, 2003). It occurs, if the Ca^2+^ level in the sieve elements that is usually extremely low (50 nM; Furch et al., 2009), surpasses a certain threshold, most likely at membrane-located Ca^2+^ hotspots in sieve elements (Hafke et al., 2009). These hotspots arise, since Ca^2+^ ions do not quickly diffuse away from the Ca^2+^ channel apertures and tend to accumulate at sites where Ca^2+^ channels are aggregated. Ca^2+^ hotspots have strong local impact. Ca^2+^ hotspots visualized in transfer cells, for instance, co-localized with cell-wall apposition giving rise to cell wall protuberances (Zhang et al., 2015). The reversibility of the forisome dispersion may depend on the activity of Ca^2+^ pumps in sieve-element membranes as observed in other cell types (Kudla et al., 2010; Huda et al., 2013).

Forisomes are conglomerates of several types of SEO-(sieve-element occlusion) proteins (Noll et al., 2007; Pélissier et al., 2008; Tuteja et al., 2010; Rüping et al., 2010; Ernst et al., 2012; Zielonka et al., 2014; Müller et al., 2014). Remarkably, no known Ca^2+^-binding motifs were found so far in legume SEOs (Tuteja et al., 2010; Srivastava et al., 2016), but the pea forisome protein PsSEO-F1 showed a cleat-cut Ca^2+^-binding capability (Srivastava et al., 2016) so that identification of Ca^2+^ binding motifs in SEO protein structures may be a matter of time.

Other members of the SEO-protein family occur in a non-crystalline filamentous form in plant families other than legumes (Rüping et al., 2010; Anstead et al., 2012). Whether these SEO proteins present as heterodimers (Anstead et al., 2012; Jekat et al., 2013) bind Ca^2+^ ions and occlude the sieve plates in an aggregated state is a matter of debate. That mass flow is not blocked by dense aggregates of SEO-proteins near the sieve plates of intact *A. thaliana* plants was taken as evidence that filamentous SEO proteins lack occlusion properties (Froelich et al., 2011). However, this conclusion seems over-hasty, because the occluding capacities of SEO proteins only come to light, when the sieve tubes are damaged. Similar experiments with damaged *A. thaliana* (Jekat et al., 2013) and tobacco (Ernst et al., 2012) plants indicate SEO-mediated sieve-plate occlusion. Moreover, aphid feeding, recorded by using the electrical penetration graph technique, showed an instantaneous interruption in response to remote burning in a range of plant species. This reaction suggests that protein-mediated sieve-tube occlusion also occurs in plant species lacking forisomes (Will et al., 2009).

Not every protein clogging event in sieve tubes is Ca^2+^ dependent. In cucurbits, phloem protein 1 (PP1) and phloem protein 2 (PP2) aggregate to insoluble polymeric plugs in response to oxidation leading to sieve-pore occlusion in cut sieve tubes (Kleinig, 1975; Alosi et al., 1988; Golecki et al., 1998). Within a couple of minutes, interaction between PP1 and PP2 seals the cut surface of cucurbit plants by exudate gelling (Clark et al., 1997; Zimmermann et al., 2013). A similar coagulation of phloem exudate was observed shortly after excision of stylets from aphids feeding on *V. faba* (Fisher and Frame, 1984). It is hard to conceive that this gelling is due to dispersing forisomes, since their number is likely to be extremely low in the exudate (none to a few). Hence, the phenomenon infers oxygen-sensitive protein gelling in broadbean sieve-tube sap as well (Arsanto, 1986).

Oxygen-stimulated linkage of PP1 and PP2 may be corroborated by Ca^2+^-binding sites. Cucurbit sieve-tubes occlude several centimeters away from a site of burning (Furch et al., 2010). The distinct decrease in soluble PP1 and PP2-dimers in sieve elements there (Furch et al., 2010) is supportive of PP1–PP2 cross-linking. Latter may depend on a longitudinal electric potential wave evoked by burning (Furch et al., 2010). The electric potential wave induces a rise in Ca^2+^ concentrations along the sieve tubes (Furch et al., 2009). PP1 is a candidate for Ca^2+^-mediated sieve-element occlusion (Furch et al., 2010) in view of its potential Ca^2+^-binding sites (McEuen et al., 1981; Arsanto, 1986). PP2 is abundantly present in the sieve-tubes of *A. thaliana* (Batailler et al., 2012), but interaction with environmental factors remains unclear. Simultaneously, the Ca^2+^ wave may enhance the level of reactive oxygen species in sieve elements (Görlach et al., 2015; Evans et al., 2016). As yet it is difficult to discriminate between the potential cues of PP1–PP2 cross-linking there. Is either the rise in Ca^2+^ or ROS or is it a collaborative action?

Obviously, sieve-tube occlusion endangers food ingestion by aphids, because their nutrients are transported by mass flow through the sieve tubes. Sequestration of Ca^2+^ ions would prevent sieve-plate occlusion and preserve mass flow. Based upon this idea, it has been postulated that aphid saliva contains Ca^2+^-binding proteins (Will and van Bel, 2006). Ca^2+^-binding properties of aphid saliva were substantiated by experiments using the *in vitro* Ca^2+^ reactivity of forisomes from *V. faba* (Knoblauch et al., 2001, 2012; Will et al., 2007). Concentrated aphid saliva, as well as EDTA, showed similar condensation effects on isolated dispersed forisomes (Will et al., 2007). It has been argued that these *in vitro* experiments using salivary probes and forisomes (Will et al., 2007) lead to an enormous overestimation of the Ca^2+^-binding capacity of saliva proteins. It is difficult to rebut this critical point using quantitative arguments. The actual saliva concentration is probably much lower in sieve tubes, punctured by one or a few aphids. On the other hand, Ca^2+^ influx brought about by a single stylet puncture is much lower than the amount of Ca^2+^ ions administered in the *in vitro* forisome experiments (Will et al., 2007). Moreover, Ca^2+^ can be readily sequestered, since saliva is being secreted near the site of puncture.

Separation by SDS-PAGE and selective staining and radio-labeling disclosed the presence of several Ca^2+^-binding proteins in the saliva of *M. viciae* (Will et al., 2007). Later proteomic analysis of saliva from *A. pisum* showed the presence of regucalcin, a Ca^2+^-binding protein involved in Ca^2+^ homeostasis (Carolan et al., 2009). Regucalcin sequesters Ca^2+^ and activates Ca^2+^ pumps (Yamaguchi, 2005). This 43-kDa protein belongs to the SMP-30 superfamily and coincides in molecular size with a 43-kDa Ca^2+^-binding protein identified by one-dimensional SDS-PAGE in the saliva of *M. viciae* (Will et al., 2007). Regucalcin was not found in the saliva of cereal aphids (Rao et al., 2013), which once again illustrates the diversity of saliva between the aphid species.

The second Ca^2+^-binding protein, detected in *A. pisum* saliva, was an ARMET protein (Wang et al., 2015a) that was identified by proteomic analysis (Carolan et al., 2011). ARMET proteins are associated with the unfolded protein response (UPR) in ER stacks (Hampton, 2000; Apostolou et al., 2008). It was argued that salivary protein concentrations in sieve-tube sap are generally low, so that Ca^2+^ sequestration by salivary ARMET should be highly localized (Wang et al., 2015a). As sieve-element wounding is likely correlated with Ca^2+^ release from ER compartments via CICR-channels (Hafke et al., 2009; Evans et al., 2016), interference with Ca^2+^ trafficking at the sieve-element ER-membranes (Hafke et al., 2009) may be a feasible option for ARMET functioning.

Recently, a third potential mode of lowering the Ca^2+^ concentration in sieve elements by salivary proteins became apparent (Sinha et al., 2016). Ca^2+^ release from the sieve-element ER stacks (Buchen et al., 1983; Sjolund and Shih, 1983; Furch et al., 2009; Hafke et al., 2009) likely contributes to increased mictoplasmic Ca^2+^ levels in response to aphid attack. Mastoparan treatments of staminal hairs of *Setcreasea purpurea* demonstrated that IP_3_-activated Ca^2+^ channels appear to be relevant for Ca^2+^ eﬄux from the ER compartments (Tucker and Boss, 1996), where the Ca^2+^ concentration is at least 10,000 times higher (Montero et al., 1995) than in the sieve-element lumen (Furch et al., 2009). As a speculation, an interference of salivary proteins with the phosphoinositide metabolism, would lead to a reduced IP_3_ production, and thus would suppress the release of Ca^2+^ ions into the sieve-element lumen. *D. noxia* biotype-2 aphids that overexpress proteins inhibiting the key enzyme phospopholipase C, relevant for IP_3_ formation, are virulent on aphid-resistant wheat plants (Sinha et al., 2016). However, it should be noted that, in contrast to overwhelming evidence for IP_3_ involvement in Ca^2+^ release in animal cells, the presence of cytosolic IP_3_ in plants has not been convincingly demonstrated as yet (Kudla et al., 2010).

The presence of Ca^2+^-binding proteins is not limited to aphids. An EF-hand motif – a helix-loop-helix structural domain in a large family of Ca^2+^-binding proteins – was found in a salivary protein of the green rice leafhopper *Nephotettix cinticeps* (Hattori et al., 2012), so that Ca^2+^-binding properties of saliva may be universal among plant sucking Hemiptera.

Ca^2+^ sequestration by salivary proteins with the objective to suppress sieve-tube occlusion may be wide-spread among aphid species. The sudden change in feeding behavior in response to remote heat shocks in diverse plant-aphid combinations (Will et al., 2009) hints at intensified saliva secretion to counteract imminent sieve-element occlusion. As a speculation, binding of Ca^2+^ by salivary proteins can be regarded as an adaptation of specialized feeders like *A. pisum* to those plants that highly rely on Ca^2+^-based occlusion. Penetration of *A. pisum* stylets did not trigger sieve-tube occlusion in *V. faba* (Walker and Medina-Ortega, 2012). This lack of reaction has been ascribed to the high specialization of pea aphids to legumes (Medina-Ortega and Walker, 2015). The saliva would sequester Ca^2+^ to such an extent that condensed forisomes are not sufficiently challenged (Will, 2016b). The latter conclusion was drawn, since feeding of generalists like *M. persicae* and *M. euphorbiae* indeed triggered forisome-mediated occlusion (Medina-Ortega and Walker, 2015). It is no surprise that Ca^2+^-channel blockers favored feeding of *M. persicae* by preventing sieve-element occlusion (Medina-Ortega and Walker, 2015), since the Ca^2+^-channel blocker gadolinium prevented forisome dispersion in sieve-element protoplasts (Hafke et al., 2007).

#### Indirect Suppression of Callose Production by Salivary Proteins?

The second slower type of sieve-tube occlusion by callose partly overlaps in time with protein occlusion (Furch et al., 2007) and is executed by β-1,3 glucan depositions around the sieve plate pores, termed “callose plugs” (van Bel, 2005). These “plugs” reside unlike protein plugs in the extracellular space and are, in fact, callose collars apposed against the cell-wall areas bordering the plasma membrane corridor that crosses the sieve pores (Evert, 1990; Ehlers et al., 2000). As result of callose deposition, the mictoplasmic corridors between sieve elements become “strangled” and phloem transport stops. It should be noted that callose is not always deposited in the extracellular space. Intracellular callose plugs of plasmodesmata are delivered by multivesicular bodies as an early step in the hypersensitive response (An et al., 2006). Furthermore, callose plugs inside plasmodesmal corridors are involved in dormancy processes (Rinne et al., 2001).

Since sieve pores are evolutionary and ontogenic “descendants” of plasmodesmata (Evert, 1977, 1990), one may expect that fundamental traits such as callose homeostasis have strong similarities between both structures and that plasmodesmal physiology might tell us much about sieve-pore physiology. Unlike the continuous callose collars in the sieve pores, plasmodesmata have a ring of callose around both neck regions (sphincters) of the cytoplasmic corridors that connect neighboring cells (Radford et al., 1998). The permanence of callose rings around the sphincters of standard plasmodesmata is a matter of debate (Levy and Epel, 2009). In sieve elements, callose depositions – necessary for the formation of sieve pores and PPUs (Evert, 1977, 1990; Barratt et al., 2011) – permanently surround the mictoplasmic corridors (through the sieve pores) between sieve elements after maturation (Ehlers et al., 2000). Plasmodesmal fluxes are inversely correlated with the degree of callose deposition (e.g., Zavaliev et al., 2011; Tilsner et al., 2016), which suggests a strong resemblance with sieve-pore functionality.

According to recent reviews (Zavaliev et al., 2011; De Storme and Geelen, 2014; Kumar et al., 2015; Tilsner et al., 2016), callose deposition results from an equilibirum imposed by the activities of plasma transmembrane β-1,3 glucan synthases or callose synthases (GSLs) and plasma-membrane anchored extracellular β-1,3 glucanases or glucan endo-1,3-hydrolases (BGs).

Callose synthases present a multigene family of large (200–220 kDa) plasma-membrane spanning proteins with both the N- and C-terminus residing in the cytoplasm (Farrokhi et al., 2006; Brownfield et al., 2007). They are typically clustered in two transmembrane regions connected by an extensive cytoplasmic loop, including an UDP-glucose catalytic site and a glysosyltransferase domain (see for reviews: De Storme and Geelen, 2014; Tilsner et al., 2016). Together with the N-terminal region, the hydrophilic loop acts as a site for interaction with regulatory molecules due to several glycosylation and phosphorylation domains (Verma and Hong, 2001). Callose synthases are probably incorporated into complex protein structures (CalS holoenzyme complexes; Amor et al., 1995; Hong et al., 2001), that include several proteins participating in the polymerization of the substrate UDP-glucose and the delivery of glucan chains into the apoplasmic space. Three genes were found to be directly associated with plasmodesmal callose deposition. Most likely, *GSL 8* is responsible for callose deposition in plasmodesmata in a wide variety of tissues (Guseman et al., 2010), *GSL7* is involved in sieve-pore shaping during sieve-element development (Barratt et al., 2011; Xie et al., 2011), and *GSL12* has a major role in adjusting the functional diameter of plasmodesmata (Vaten et al., 2011). Their distribution over the cell types seems to indicate that all three genes are engaged in callose homeostasis in sieve elements.

Glucanases are the functional counterpart of callose synthases. In *Arabidopsis*, the glucanase family comprises 50 representatives of β-1,3-glucan-degrading enzymes (Doxey et al., 2007). Thus far, three of them were found to be associated with plasmodesmal regions and were characterized: one in leaves (Zavaliev et al., 2013) and two in roots (Benitez-Alfonso et al., 2013). They are likely anchored by a C-terminal glycophosphatidylinositol (Gaudioso-Pedraza and Benitez-Alfonso, 2014) to the outer leaflet of the plasma membrane and thus face the inner side of the cell wall. β-1,3-glucanases are produced in the Golgi system and secreted into the apoplasmic space via exocytosis (De Storme and Geelen, 2014). In conclusion, it is of paramount importance to note that the regulation of callose deposition is located in entirely different cell compartments. Callose synthases operate in the cytosol, glucanases in the extracellular space.

Pioneer experiments of Erwee and Goodwin (1983) demonstrated that the plasmodesmal diameter is under the control of Ca^2+^ ions. A rise in Ca^2+^ concentration, probably from the ER stores (Tucker, 1988; Tucker and Boss, 1996), coincides with enhanced callose deposition (Tucker and Boss, 1996; Holdaway-Clarke et al., 2000) and, hence, increased obstruction of symplasmic transports (Sager and Lee, 2014). As a result, abrupt changes in intercellular Ca^2+^ concentration have an immediate effect on intercellular communication. The molecular mechanisms that control this crucial Ca^2+^ effect on callose synthesis has yet to be thoroughly investigated (Zavaliev et al., 2011; De Storme and Geelen, 2014; Kumar et al., 2015; Tilsner et al., 2016).

Similar Ca^2+^-controlled mechanisms are supposed to regulate mass flow through sieve tubes (Kauss, 1987). The uneven deployment of Ca^2+^ channels along sieve elements renders credence for a strong relationship between Ca^2+^ level and callose deposition (Furch et al., 2009). The modified plasmodesmata of sieve elements (sieve pores and pore-plasmodesm units or PPUs, the symplasmic connections with the companion cells) possess constitutive callose linings (Evert, 1990; Ehlers et al., 2000). Sieve pores and PPUs perfectly co-localize with Ca^2+^ hotspots (Furch et al., 2009; Hafke et al., 2009; see section Ca^2+^-binding proteins for a hotspot definition). Aggregates of Ca^2+^ channels along the sieve-element plasma membrane allow changes in Ca^2+^ level focused at sites, where they are physiologically relevant for immediate action such as callose deposition and protein dispersion (Hafke et al., 2009). Such a phenomenon is by no means unique. In developing transfer cells, cytosolic Ca^2+^ plumes co-localize with the deposition of cell-wall protrusions, which may consist in part of callose to provide a plastic matrix for embedment of stiffer cell-wall components (Zhang et al., 2015).

In disturbed, yet undamaged sieve elements, supplementary callose depositions at sieve pores and PPUs disappear within a couple of hours after disturbance (Furch et al., 2007, 2010). The quick build-up and slower breakdown of callose in sieve elements raises questions regarding the control mechanisms. The basic level of callose deposition may be balanced by the counteraction of callose synthases and glucanases (Zavaliev et al., 2011; De Storme and Geelen, 2014; Kumar et al., 2015).

The rapidity of callose build-up in response to wounding (seconds to minutes) suggests a regulation on the protein level. Ca^2+^ released into the sieve element mictoplasm may readily bind to the CalS complex (Hong et al., 2001) with the putative participation of ATP and calmodulin (Levy and Epel, 2009) under favorable redox conditions (see Zavaliev et al., 2011). As a speculation, a rapid rise in intracellular Ca^2+^ will boost the activity of the callose synthases to an extent that cannot be counteracted by glucanase activity and additional callose will be deposited. If however, Ca^2+^ is removed from the local cytosol to the ground level as will happen in undamaged sieve elements (Furch et al., 2007), glucanases have the chance to reduce the callose layer to the original thickness in sieve elements.

It is possible that callose synthases and glucanases are upregulated at the transcript level during peaks of callose turnover. It should be realized, however that the situation in enucleate sieve elements differs radically from that in nucleate cells. Gene expression of callose synthases and glucanases must take place in cells adjacent to the sieve elements, most probably the companion cells, from which the glucanases are released into the apoplasmic space by exocytosis (De Storme and Geelen, 2014).

In conclusion, the previous considerations infer altogether at least two potential modes of callose suppression by aphid saliva. As callose sealing is considered to be a general, but quantitatively species-specific (Saheed et al., 2009) defense mechanism against aphids and other sucking insects (Hao et al., 2008), Ca^2+^ sequestration by salivary proteins may enable the aphids to prevent callose deposition by suppression of Ca^2+^-stimulated callose-synthase activity. When thick callose depositions are found in sieve tubes as a response to aphid attack, Ca^2+^ sequestration has likely been insufficient to prevent callose synthesis (Saheed et al., 2009). Ca^2+^-binding proteins may also disturb the equilibrium between callose build-up and degradation so that stimulation of glucanase action is conceivable. Breakdown of callose depositions by salivary glucanases is unlikey, since they would be introduced into the luminal compartment of sieve elements, out of reach of the callose located in the cell-walls. It is much more logical; therefore, that callose breakdown is facilitated by upregulation of plant glucanases (Van der Westhuizen et al., 1998) probably making use of salivary effectors. In keeping with this idea, infestation with *R. padi* induced a transcript abundance of three β-1,3-glucanases in 15 barley breeding lines (Mehrabi et al., 2016). In Mp55-expressing *Arabidopsis* plants, callose deposition in response to aphid infestation is reduced (Elzinga et al., 2014), but the molecular action of Mp55 is yet to be identified.

#### Proteases

The presence of proteases in the alimentary tract of aphids is an established fact (Rahbé et al., 1995; Cristofoletti et al., 2003, 2006; Pyati et al., 2011). It was suspected for a long time that aphid saliva as well contains several proteases, although the first functional assays were not successful (Cherqui and Tjallingii, 2000). Some proteases were recently identified using proteomics on aphid saliva and salivary gland extracts of *A. pisum* (Carolan et al., 2009, 2011). In mammalian systems, the M1 zinc metalloprotease and M2 metalloproteases (angiotensin-converting enzymes, ACEs; Wang et al., 2015b) bind zinc at their catalytic domain (Lausten et al., 2001; Kim et al., 2003), while Ca^2+^ regulates their catalytic activity (Goto et al., 2007). ACEs remove dipeptides from short oligopeptides (Ehlers and Riordan, 1989) which contrasts the action of M1 metalloproteases cleaving the terminal amino acids from proteins (Itturioz et al., 2001; Naqvi et al., 2005). It is not excluded that salivary proteases are species-specific in aphids: the metalloproteases detected in *A. pisum* (Carolan et al., 2009, 2011) were not found in the saliva of *S. avenae* and *M. dirhodum* (Rao et al., 2013). Like Ca^2+^-binding proteins, metalloproteases were also found in the saliva of other insect groups such as the phytophagous thrips *Frankliniella occidentalis* (Stafford-Banks et al., 2014) and the blood-feeding tick *Ixodes scapularis* (Francischetti et al., 2003; Decrem et al., 2008).

The putative proteolytic activity of saliva was verified by two functional assays (Furch et al., 2015). Albumin was degraded by salivary probes of *A. pisum* in the presence or absence of EDTA. Rapid albumin breakdown, in particular in the absence of EDTA, indicated the involvement of metalloproteases in protein degradation (Furch et al., 2015). Mixing salivary probes of *A. pisum* and *M. euphorbiae*, respectively, with sieve-tube exudate of *Cucurbita maxima* demonstrated that PP1 and PP2 in the protein-rich exudate were degraded with time (Furch et al., 2015). This is supportive of protease-mediated breakdown of sieve-element proteins by salivary proteases. PP1, the protein specialized in occlusion, was more rapidly broken down than PP2, which appeared to be recalcitrant to breakdown by proteases in the alimentary tract (Rahbé et al., 1995). In conclusion, proteases may act as an auxiliary tool to remove proteinaceous occlusions.

A third functional aspect of protease activity pertains to aphid nutrition (Carolan et al., 2009; Furch et al., 2015). As sieve-tube sap is poor in several essential amino acids (Gündüz and Douglas, 2009), aphids are mostly short of these indispensable compounds. The deficiency is compensated by endosymbiotic bacteria that transform non-essential into essential amino acids (Baumann et al., 1995). Amino acids from degraded proteins in sieve tubes could enhance the aphid’s diet quality. The breakdown strategy may be more successful in dicots than in monocots given the higher protein contents in sieve-tube sap of dicots (2–100 mg ml^-1^: Richardson et al., 1982; Schobert et al., 1998; Zimmermann et al., 2013) as compared to monocots (0.1–1.0 mg ml^-1^: Fisher et al., 1992; Schobert et al., 1998; Gaupels et al., 2008).

It is unclear, if protein breakdown by salivary proteases merely has a non-selective character as indicated by *in vitro* breakdown of proteins by salivary proteases (Furch et al., 2015). In view of the presence of so many other vital proteins in the saliva, however, additional selective protein-breakdown machinery seems logical. Selective protease activity is usually associated with ubiquitin tagging accompanied by several accessory enzymes (van der Hoorn, 2008) and the final transfer of the ubiquitin-protein complex to the proteasome. The question arises, whether ubiquitin as a first indicator of selective protein degradation occurs in sieve elements or aphid saliva. After the first detection of ubiquitin in *Ricinus communis* sieve-tube exudate (Schobert et al., 1995), proteomics disclosed the presence of at least 116 components involved in proteasome-associated protein degradation in the sieve-tube sap of cucurbits (Lin et al., 2009) This indication for a complete proteolytic system in sieve elements was consistent with the presence of ubiquination compounds in the sieve-tube sap of rice (Aki et al., 2008) and rapeseed (Giavalisco et al., 2006) and the likeliness of proteasomes in sieve elements (Ingvardsen and Veierskov, 2001). In contrast, native ubiquitin nor proteasomal components have not been found in aphid saliva thus far (Chaudhary et al., 2015). Should selective protein degradation be executed by salivary proteases, they likely make use of the sieve-element breakdown machinery.

#### Detoxifying Proteins

The presence of polyphenoloxidases (Peng and Miles, 1988; Miles and Oertli, 1993; Madhusudhan and Miles, 1998; Urbanska et al., 1998; Cherqui and Tjallingii, 2000; Harmel et al., 2008; Cooper et al., 2011; Chaudhary et al., 2015) and peroxidases in saliva (Miles and Peng, 1989; Cherqui and Tjallingii, 2000; Chaudhary et al., 2015) is interpreted as a reductive weapon against plant phenols and reactive oxygen species (Leszczynski et al., 1985; Moloi and van der Westhuizen, 2006). A similar function has been attributed to oxidoreductases (Miles and Oertli, 1993) and GMC-oxidoreductases (Carolan et al., 2009; Nicholson et al., 2012; Rao et al., 2013). Given the differences in phenol localization (epidermis, cortex, mesophyll, sieve tubes) and phenol quantities between plant species, some of the oxidases may be most effective against toxic substances along the stylet pathway and others against phenols or other toxic substances residing inside the sieve tubes. Peroxidases may also play a role in the breakdown of hydrogen peroxide, a booster of Ca^2+^-channel gating (Lecourieux et al., 2006), and hence may reduce the occlusion of sieve tubes. The same may be true for GMC-oxidoreductases, which possess ROS (reactive oxygen species)-scavenging properties. However, the low pH optima of these enzymes raise some reserve as for their efficiency in view of the alkaline sieve-tube milieu (Will, 2016b). The ROS-scavengers possibly interact with the native ROS-scavenging system in sieve tubes (Walz et al., 2002).

Evidence has emerged that the redox status impacts on callose synthesis. In *Arabidopsis* mutants defective in the thioredoxin-m3 gene (*TRX-m3*), GFP diffusion out of the sieve elements was reduced in comparison with wild-type plants. These mutants accumulated ROS and contained higher levels of callose in root tips (Benitez-Alfonso et al., 2009). Overexpression of TRX-m3, by contrast, resulted in enhanced intercellular transport compared to wild-type plants. Moreover, reduced macromolecular trafficking in wild-type plants treated with oxidants (Benitez-Alfonso et al., 2009) and in *Arabidopsis* mutants defective in the production of glutathione (Cairns et al., 2006) inferred that oxidants stimulate callose production. Consequently, oxidoreductases and other ROS scavengers in aphid saliva may limit the cellular damage caused by ROS (e.g., lipids and proteins), but also impair callose production by withdrawal of oxidants. This conclusion may be premature as in *Arabidopsis ise1* mutants in which the ROS level exceeds that in wild-type plants, intercellular transport is stimulated (Stonebloom et al., 2009). It seems beyond doubt that redox homeostasis regulates intercellular trafficking probably by interaction with callose metabolism. It is unclear, whether ROS directly act upon callose deposition or if Ca^2+^ channels are gated by ROS as demonstrated for hydrogen peroxide (Lecourieux et al., 2006).

Salivary dehydrogenases and glucose oxidases possibly interfere with plant defense systems regulated by jasmonic acid (JA) and salicylic acid (SA) during aphid infestation (Louis and Shah, 2013). The suppressed JA-controlled defense responses of *A. thaliana* to *Brevicoryne brassicae* infestation (Kusnierczyk et al., 2011) could be due to a diminished production of JA (Takemoto et al., 2013) conferred by the above-mentioned enzymes. It is unclear, if the aphid-imposed modulation of genes engaged in SA-synthesis (Zhu-Salzman et al., 2004) depends on the cross-talk with the JA pathway (Pieterse et al., 2012) or on so-called salivary effectors.

Chaudhary et al. (2015) detected several other defense modulators in the saliva of *M. euphorbia*, mainly interfering with regulatory enzymes of the oxidative burst, a hallmark in plant immunity. In addition, lipase-like proteins in the same secretome may function as virulence factors to promote aphid colonization (Chaudhary et al., 2015).

#### Effector Proteins of Unknown Function

In contrast to the protein classes discussed above, the majority of salivary proteins may not interfere in a direct disruptive manner with events in cell walls or inside sieve elements. They probably interact in an unknown fashion with the protein network involved in plant defense. Effectors act in a broad spectrum of pathogens and are generally defined as molecules that alter function and/or structure of host cells (Hogenhout et al., 2009). This definition includes a group of proteins that effect on aphid fecundity and behavior. Their effect on suppression or elicitation of plant defense, respectively, are inferred from the rates of aphid colonization or fecundity (Bos et al., 2010; Pitino et al., 2011; Atamian et al., 2013; Elzinga and Jander, 2013; Pitino and Hogenhout, 2013; Rodriguez and Bos, 2013; Elzinga et al., 2014; Rodriguez et al., 2014; Chaudhary et al., 2015).

As a first salivary protein, C002 was termed “effector”. C002 discovered in the saliva of *A. pisum* and is required for continuous aphid feeding (Mutti et al., 2008). When C002 is transiently overexpressed in plants that were subsequently infested by *M. persicae*, aphid fecundity is increased (Bos et al., 2010). In reverse, suppression of C002 expression by RNA interference is lethal in *A. pisum* (Mutti et al., 2006) and causes reduced fecundity in *M. persicae* (Pitino et al., 2011). Interestingly, C002 is only required for feeding on plants, not on diets (Mutti et al., 2008), which implies a role in compatibilty. C002 may have significance for the host range of an aphid species. The ability of *M. persicae* to feed on a broad range of host plants may be due to the presence of a repeated 7-amino acid motif in C002 of *M. persicae* that is absent in C002 of *A. pisum* (Pitino and Hogenhout, 2013).

Aphid colonization is also promoted by two further salivary proteins detected in *M. persicae* (Pitino and Hogenhout, 2013). These effector proteins [PIntO1, (Mp1) and PintO2 (Mp2)] are acting in a plant species-specific manner (Pitino and Hogenhout, 2013). The effector Mp55 also suppresses plant defense. Mp55-expressing *A. thaliana* plants were more attractive to aphids in choice assays, whereas Mp55 silencing in aphids led to reduced reproduction rates (Elzinga et al., 2014).

Likewise, saliva of *M. euphorbiae* contains two proteins, Me10 and Me23 that promote aphid colonization, as they seem to suppress plant defense when expressed in *Nicotiana benthamiana* (Atamian et al., 2013). Me23 possesses a conserved glutathione peroxidase domain and might interfere with plant defense responses, while the function of Me10 is unknown (Chaudhary et al., 2015). They are both phosphorylated, but the molecular significance of these phosphorylation sites is elusive (Chaudhary et al., 2015). When expressed in tomato, only Me10 increased aphid fecundity underlining once again the species-specificity of effectors (Atamian et al., 2013), the more as expression of the Me10-homologue Mp58 resulted in a decreased fecundity in *Nicotiana tabacum* and *Arabidopsis* (Elzinga et al., 2014). As for the function of these proteins, the fundamental question remains as whether the increased fecundity of aphids is due to the suppression of plant defense or to a promoted efficiency of aphid feeding.

By contrast, some salivary proteins such as Mp10 and Mp42 from *M. persicae* suppress aphid reproductive performance and appear to elicit plant defense reactions (Bos et al., 2010). *In planta* overexpression of Mp10 and Mp42 in *N. benthamiana* reduced aphid feeding (Bos et al., 2010). Further work revealed that Mp10 and Mp42 are engaged in elicitation of plant defense in distinct ways at different subcellular locations. Transient overexpression activated JA and SA signaling pathways, while Mp42 did not (Rodriguez and Bos, 2013; Rodriguez et al., 2014).

Recently, a macrophage migration inhibitory factor (MIF) was identified in the saliva of *A. pisum* using 2D-DIGE-MALDI-TOF/MS (Vandermoten et al., 2014; Naessens et al., 2015) that sheds some light on effector action. In mammals MIFs are pro-inflammatory cytokins that regulate immune responses (Calandra and Roger, 2003). Of five members of a MIF multigene family in *A. pisum* (Dubreuil et al., 2014), just one (*ApMIF1*) is present in the saliva (Naessens et al., 2015; Reymond and Calandra, 2015). This protein was postulated to suppress the immune response of plants by inhibiting the expression of defense-related genes (Naessens et al., 2015).

#### Endosymbiont-Derived Proteins

Beside proteins that possess a secretory signal sequence and were classified as salivary components, a number of proteins that have been identified in salivary glands are lacking such a sequence (Carolan et al., 2011). It has been a matter of dispute, if such proteins belong to the salivary proteome. Recently, eleven proteins from *Buchnera aphidicola* proteins were detected in the saliva of *M. euphorbia*, four of which were attributed to gel saliva only (Chaudhary et al., 2014). One of the identified proteins was the chaperone GroEL, a heat shock protein engaged in protein folding, which is abundant in *B. aphidicola* (Baumann et al., 1996). Several GroEL expression studies in plants demonstrated that GroEL is recognized intra- and extracellularly and functions as a microbe associated molecular pattern (MAMP) triggering an oxidative burst and the expression of a number of immunity marker genes (Chaudhary et al., 2014). This suggests that GroEL has to be regarded as a biologically relevant contaminant that elicits plant defense. Aphids reacted to increased GroEL expression by diminished fecundity (Chaudhary et al., 2014; Elzinga et al., 2014). The investigators interpreted the plant defense response as being targeted to *B. aphidicola*, which impacts negatively on the reproduction of the aphids (Chaudhary et al., 2014). Removal of *B. aphidicola* activity indeed led to a delayed aphid development and a considerable decrease of reproduction rates (Sasaki et al., 1991).

It is of major importance to investigate the route followed by GroEL into the salivary glands. Such studies may give a clue, as if proteins without a secretory sequence are produced by the gland cells or are imported into the glands after being produced by other organs.

### Impact of Chitin Fragments in Aphid Saliva on Plant Defense Responses?

Although chitin does not belong to the salivary secretome, aphid saliva may contain chitin fragments. They may be rubbed away by mechanical stress during stylet movement or are liberated by plant chitinases. In the presence of chitin, the lysin domain-containing glycophosphatidylinositol-anchored protein 2 (LYM2) which is an *Arabidopsis* homologue of a chitin receptor-like protein (Kaku et al., 2006), becomes involved in the reduction of plasmodesmal traffic (Faulkner et al., 2013). As a speculation, chitin loss from the stylet surface may impact on the cellular response to stylet penetration by callose deposition, mediated by the plasmodesma-located LYM2-protein. Thus, chitin fragments may act as pathogen-associated molecular patterns that strengthen the plant-defense response. It should be noted, however, that CERK1 (CHITIN ELECITOR RECEPTOR KINASE1) does not seem to be engaged in chitin recognition (Prince et al., 2014).

## Concluding Remarks

Unequivocal separation of gel and watery saliva is an absolute prerequisite for the functional assessment of salivary proteins, but suffers from cross-contamination in dietary solutions up till now. Analysis of proteins collected in specific diets (e.g., pH 5 or 7) or *in aera* will enable a distinct separation of the saliva types. It should be taken into account that salivary protein patterns obtained with artificial diets and plants often differ.

Protein profiles of watery saliva exhibit large interspecific variations. The evolutionary challenges imposed by the highly variable symplasmic conditions between plant species appear to have had a strong impact on the nature of the salivary proteins. The protein profiles of watery saliva indicate that there is a wealth of possibilities for interaction between host plant and aphid species.

The protein composition of gel saliva is anticipated to be more conservative in view of the lower interspecific variability of the cell-wall milieu.

Studies point to an adaptation of the salivary protein composition to the plant host, but more research is required, i.e., on the time-dependence and on the transgenerational nature of the adaptation.

The proven and putative functions of (putative) salivary proteins are in keeping with the stylet itinerary and the proposed mode of orientation. Several functional groups of salivary proteins have been distinguished or postulated thus far:

(1) Proteins that provide the structural backbone for the salivary sheath.
(2) Proteins that degrade cell-wall carbohydrates and by doing so facilitate stylet movement and give rise to the production of pathogen-induced molecular patterns.
(3) Proteins that function in prevention or degradation of sieve-plate occlusion (by proteins and callose) by sequestration of Ca^2+^ ions, in Ca^2+^ homeostasis, and in triggering several signaling cascades under the control of Ca^2+^ ions.
(4) Proteins engaged in proteolysis, which provide supplementary supply of organic N-compounds to the aphid diet, in the degradation of protein plugs on sieve plates, or in the sabotage of protein-mediated plant defense mechanisms.
(5) Proteins that regulate ROS levels, which in turn are associated with local signaling cascades or are engaged in processes that trigger long-distance signals and distant signaling cascades.
(6) Proteins involved in detoxification of a variety of poisonous compounds such as phenols.
(7) Proteins denominated simply “effectors” with an unknown involvement in host-plant defense responses, but with a clear impact on aphid fitness, e.g., fecundity. Some have phosphorylation traits, others suppress immune responses of host plants.
(8) Other salivary components such as endosymbiont-derived proteins interfere with the protein-mediated interaction between aphids and host plants.

With the aid of their vast arsenal of salivary proteins, aphids trigger and suppress plant defense in parallel. If and how activation and suppression of plant defense go hand in hand and if aphids benefit from a local induction of plant defense, remains to be investigated. Identification of salivary proteins of interest by use of proteomics is the first step. The search for their functional and, hence, biological relevance is the next essential step.

## Author Contributions

The authors made equal intellectual contributions to the work and approved it for publication.

## Conflict of Interest Statement

The authors declare that the research was conducted in the absence of any commercial or financial relationships that could be construed as a potential conflict of interest.

## References

[B1] Abdellatef E. Will T. Koch A. Imani J. Vilcinskas A. Kogel K.-H. (2015). Silencing the expression of the salivary sheath protein causes transgenerational feeding suppression in the aphid *Sitobion avenae*. *Plant Biotech. J.* 13 849–857. 10.1111/pbi.12322 25586210

[B2] Adams J. B. Drew M. E. (1965). A cellulose-hydrolyzing factor in aphid saliva. *Can. J. Zool.* 43 489–496. 10.1139/z65-048 14314950

[B3] Aki T. Shigyo M. Nakano R. Yoneyama T. Yanagisawa S. (2008). Nano scale proteomics revealed the presence of regulatory proteins including three FT-like proteins in phloem and xylem saps from rice. *Plant Cell Physiol.* 49 767–790. 10.1093/pcp/pcn049 18372294

[B4] Alosi M. C. Melroy D. L. Park R. D. (1988). The regulation of gelation of phloem exudates from *Cucurbita* fruit by dilution, glutathione and gluthatione reductase. *Plant Physiol.* 86 1089–1094. 10.1104/pp.86.4.1089 16666036 PMC1054632

[B5] Amor Y. Haigler C. H. Johnson S. Wainscott M. Delmer D. P. (1995). A membrane-associated form of sucrose synthase and its potential role in synthesis of cellulose and callose in plants. *Proc. Natl. Acad. Sci. U.S.A.* 92 9353–9357. 10.1073/pnas.92.20.9353 7568131 PMC40983

[B6] An Q. Ehlers K. Kogel K.-H. van Bel A. J. E. Hückelhoven R. (2006). Multivesicular compartments proliferate in susceptible and resistant MLA12-barley leaves in response to infection by the biotrophic powdery mildew fungus. *New Phytol.* 172 563–576. 10.1111/j.1469-8137.2006.01844.x 17083686

[B7] Anstead J. A. Froehlich D. R. Knoblauch M. Thompson G. A. (2012). *Arabidopsis* P-protein filament formation requires both ATSEO1 and AtSEO2. *Plant Cell Physiol.* 53 1089–1094. 10.1093/pcp/pcs046 22470058

[B8] Apostolou A. Shen Y. Liang Y. Luo J. Fang S. (2008). Armet, a UPR-upregulated protein, inhibits cell proliferation and ER stress-induced cell death. *Exp. Cell Res.* 314 2454–2467. 10.1016/jexcr.2008.05.001 18561914 PMC6719340

[B9] Arsanto J. P. (1986). Ca2+ binding sites and phosphatase activities in sieve element reticulum and P-protein of chick-pea phloem. A cytochemical and X-ray microanalysis survey. *Protoplasma* 132 160–171. 10.1007/bf01276996

[B10] Atamian H. S. Chaudhary E. Dal Cin V. Bao E. Girke T. Kaloshian I. (2013). In planta expression of potato aphid *Macrosiphon euphorbiae* effectors Me10 and Me23 enhances aphid fecundity. *Mol. Plant Microbe Interact.* 26 67–74. 10.1094/MPMI-06-12-0144-Fl 23194342

[B11] Auclair J. L. (1969). Nutrition of plant-sucking insects on chemically defined diets. *Entomol. Exp. Appl.* 12 623–641. 10.1111/j.1570-7458.1969.tb02558.x

[B12] Aziz A. Gauthier A. Bézier A. Poinssot B. Joubert J. M. Pugin A. (2007). Elicitor and resistance-inducing activities of β-1,4 cellodextrins in grapevine, comparison with β-1,3 glucans and α-1,4 oligogalacturonides. *J. Exp. Bot.* 58 1463–1472. 10.1093/jxb/erm008 17322548

[B13] Backus E. A. McLean D. L. (1985). Behavioral evidence that the precibarial sensilla of leafhoppers are chemosensory and function in host discrimination. *Entom. Exp. Appl.* 37 219–228. 10.1111/j.1570-7458.1985.tb03478.x

[B14] Barratt D. H. P. Kölling K. Graf A. Pike M. Calder G. Findlay K. (2011). Callose synthase GLS7 is necessary for normal phloem transport and inflorescence growth in *Arabidopsis*. *Plant Physiol.* 155 328–341. 10.1104/pp.110.166330 21098675 PMC3075753

[B15] Batailler B. Lemaitre T. Vilaine F. Sanchez C. Renard D. Cayla T. (2012). Soluble and filamentous proteins in *Arabidopsis* sieve elements. *Plant Cell Environ.* 35 1258–1273. 10.1111/j.1365-3040.2012.02487.x 22292537

[B16] Bates G. W. Goldsmith M. H. M. Goldsmith T. H. (1983). Separation of tonoplast and plasma membrane potential and resistance in cells of oat coleoptiles. *J. Membrane Biol.* 66 15–23. 10.1007/bf01868478

[B17] Baumann P. Baumann L. Clark M. A. (1996). Levels of *Buchnera aphidicola* chaperonin GroEL during growth of the aphid *Schizaphis graminum*. *Curr. Microbiol.* 32 279–285. 10.1007/s002849900050

[B18] Baumann P. Baumann L. Lai C.-Y. Rouhbakhsh D. Moran N. A. Clark M. A. (1995). Genetics, physiology, and evolutionary relationships of the genus *Buchnera*: intracellular symbionts of aphids. *Annu. Rev. Microbiol.* 49 55–94. 10.1146/annurev.mi.49.100195.000415 8561471

[B19] Benitez-Alfonso Y. Cilia M. San Roman A. Thomas C. Maule A. Hearn S. (2009). Control of *Arabidopsis* meristem development by thioredoxin-dependent regulation of intercellular transport. *Proc. Natl. Acad. Sci. U.S.A.* 106 3615–3620. 10.1073/pnas.0808717106 19218459 PMC2651306

[B20] Benitez-Alfonso Y. Faulkner C. Pendle A. Miyashima S. Helariutta Y. Maule A. (2013). Symplastic intercellular connectivity regulates lateral root patterning. *Dev. Cell* 26 136–147. 10.1016/j.devcell.2013.06.010 23850190

[B21] Bethmann B. Simonis W. Schönknecht G. (1998). Light induced changes of cytosolic pH in *Eremosphaera viridis*: recordings and kinetic analysis. *J. Exp. Bot.* 49 1129–1137. 10.1093/jexbot/49.324.1129

[B22] Bos J. I. B. Prince D. Pitino M. Maffei M. E. Win J. Hogenhout S. A. (2010). A functional genomics approach identifies candidate effectors from the aphid species *Myzus persicae* (green peach aphid). *PLoS Genetics* 6:e1001216 10.1371/journal.pgen.1001216 PMC2987835 21124944

[B23] Brownfield L. Ford K. Doblin M. S. Newbigin E. Read S. Bacic A. (2007). Proteomic and chemical evidence links the callose synthase in *Nicotiana alata* pollen tubes to the product of the NAGSL1 gene. *Plant J.* 52 147–156. 10.1111/j.1365-313x.2007.03219.x 17666022

[B24] Buchen B. Hensel D. Sievers A. (1983). Polarity in mechanoreceptor cells of trigger hairs of *Dionaea muscipula* Ellis. *Planta* 158 458–468. 10.1007/bf00397740 24264856

[B25] Cairns N. G. Pasternak M. Wachter A. Corbett C. S. Meyer A. J. (2006). Maturation of *Arabidopsis* seeds is dependent on glutathione biosynthesis within the embryo. *Plant Physiol.* 141 446–455. 10.1104/pp.106.077982 16531482 PMC1475471

[B26] Calandra T. Roger T. (2003). Macrophage migration inhibitory factor: a regulator of innate immunity. *Nat. Rev. Immunol.* 3 791–800. 10.1038/nri1200 14502271 PMC7097468

[B27] Campbell D. C. Dreyer D. L. (1985). Host-plant resistance of sorghum: differential hydrolysis of sorghum pectic substances by polysaccharases of greenbug biotypes (*Schizaphis graminum*, Homoptera: Aphididae). *Arch. Insect Biochem. Physiol.* 2 203–215. 10.1002/arch.940020208

[B28] Carolan J. C. Caragea D. Reardon K. T. Mutti N. S. Dittmer N. Pappan K. (2011). Predicted effector molecules in the salivary secretome of the pea aphid (*Acyrthosiphon pisum*): a dual transcriptomic/proteomic approach. *J. Proteome Res.* 19 1505–1518. 10.1021/pr100881q 21226539

[B29] Carolan J. C. Fitzroy C. I. J. Ashton P. D. Douglas A. E. Wilkinson T. L. (2009). The secreted salivary proteome of the pea aphid *Acyrthosiphon pisum* characterised by mass spectrometry. *Proteomics* 9 2457–2467. 10.1002/pmic.200800692 19402045

[B30] Chaudhary R. Atamian H. S. Shen Z. Briggs S. P. Kaloshian I. (2015). Potato aphid salivary proteome: enhanced salivation using resorcinol and identification of aphid phosphoproteins. *J. Proteome Res.* 14 1762–1778. 10.1021/pr501128k 25722084

[B31] Chaudhary R. Atamian H. S. Shen Z. X. Briggs S. P. Kaloshian I. (2014). GroEL from the endosymbiont *Buchnera aphidicola* betrays the aphid by triggering plant defense. *Proc. Natl. Acad. Sci. U.S.A.* 111 8919–8924. 10.1073/pnas.1407687111 24927572 PMC4066539

[B32] Cherqui A. Tjallingii W. F. (2000). Salivary proteins of aphids, a pilot study on identification, separation, and immunolocalisation. *J. Insect Physiol.* 46 1177–1186. 10.1016/s0022.1910(00)00037-8 10818245

[B33] Clark A. M. Jacobsen K. R. Bostwick D. E. Dannenhoffer J. M. Skaggs M. I. Thompson G. A. (1997). Molecular characterization of a phloem-specific gene encoding the filament protein, phloem protein 1 (PP1), from *Cucurbita maxima*. *Plant J.* 12 49–61. 10.1046/j.1365-313X.1997.12010049.x 9263452

[B34] Cooper W. R. Dillwith J. W. Puterka G. J. (2010). Salivary proteins of Russian wheat aphid (Hemiptera: Aphididae). *Environ. Entom.* 39 223–231. 10.1603/EN09079 20146860

[B35] Cooper W. R. Dillwith J. W. Puterka G. J. (2011). Comparisons of salivary proteins from five aphid (Hemiptera: Aphididae) species. *Environ. Entom.* 40 151–156. 10.1603/EN10153 22182624

[B36] Cosgrove D. J. (2005). Growth of the plant cell wall. *Nat. Rev. Mol. Cell Biol.* 6 850–861. 10.1038/nrml1746 16261190

[B37] Cristofoletti P. T. Rahbé Y. Terra W. R. (2006). Characterisation of a membrane-bound aminopeptidase purified from *Acyrthosiphon pisum* midgut cells. *FEBS J.* 273 5574–5588. 10.1111/j.1742-4658.2006.05547.x 17212776 PMC7164072

[B38] Cristofoletti P. T. Ribeiro A. F. Deraison C. Rahbé Y. Terra W. (2003). Midgut adaptation and digestive enzyme distribution in a phloem feeding insect, the pea aphid *Acyrthosiphon pisum*. *J. Insect Physiol.* 49 11–24. 10.1016/S0022-1910(02)00222-6 12770012

[B39] Cui N. Yang P. C. Guo K. Kang L. Cui F. (2016). Large-scale gene expression reveals different adaptations of *Hyalopterus personikus* to winter and summer host plants. *Insect Sci.* 10.1111/1744-7917.12336 28547891

[B40] De Storme N. Geelen D. (2014). Callose homeostasis at plasmodesmata: molecular regulators and developmental relevance. *Front. Plant Sci.* 5:138 10.3389/fpls.00138 PMC4001042 24795733

[B41] Decrem Y. Beaufays J. Blasiolu V. Lahaye K. Brossard M. Vanhamme L. (2008). A family of putative metalloproteases in the salivary glands of the tick *Ixodes ricinus*. *FEBS J.* 275 1485–1499. 10.1111/j.1742-4658.2008.06308.x 18279375

[B42] Demidchik V. Maathuis F. J. M. (2007). Physiological roles of non-selective cation channels in plants: from salt stress to signalling and development. *New Phytol.* 175 387–404. 10.1111/j.1469-8137.2007.2128.x 17635215

[B43] Dinant S. Lucas W. J. (2013). “Sieve elements: puzzling activities deciphered through proteomic studies,” in *Phloem. Molecular Cell Biology, Systemic Communication, Biotic Interactions*, eds Thompson G. A. van Bel A. J. E. (Chichester: Wiley-Blackwell), 157–185.

[B44] Doxey A. C. Yaish M. W. F. Moffatt B. A. Griffith M. McConkey B. J. (2007). Functional divergence in the *Arabidopsis* beta-1,3-glucanase family inferred by phylogenetic reconstruction of expression states. *Mol. Biol. Evol.* 24 1045–1055. 10.1093/molbev/msm024 17272678

[B45] Dubreuil G. Deleury E. Crochard D. Simon J.-C. Coustau C. (2014). Diversification of MIF immune regulators in aphids: link with agonistic and antagonistic interactions. *BMC Genomics* 15:762 10.1186/1471.2164.15.762 PMC4169804 25193628

[B46] Ehlers K. Knoblauch M. van Bel A. J. E. (2000). Ultrastructural features of well-preserved and injured sieve elements; minute clamps keep the phloem transport conduits free for mass flow. *Protoplasma* 214 80–92. 10.1007/BF02524265

[B47] Ehlers M. R. Riordan J. F. (1989). Angiotensin-converting enzyme: new concepts concerning its biological role. *Biochemistry* 28 5311–5318. 10.1021/bi00439a001 2476171

[B48] Elzinga D. A. de Vos M. Jander G. (2014). Suppression of plant defenses by a *Myzus persicae* (green peach aphid) salivary effector protein. *Mol. Plant Microbe Interact.* 27 747–756. 10.1094/MPMI-01-14-0018-R 24654979 PMC4170801

[B49] Elzinga D. A. Jander G. (2013). The role of protein effectors in plant-aphid interactions. *Curr. Opin. Plant Biol.* 16 451–456. 10.1016/j.pbi.2013.06.018 23850072

[B50] Ernst A. M. Jekat S. B. Zielonka S. Müller B. Neumann U. Rüping B. (2012). Sieve element occlusion (SEO) genes encode structural proteins involved in wound sealing of phloem. *Proc. Natl. Acad. Sci. U.S.A.* 109 1980–1989. 10.1073/pnas.1202999109 PMC3396537 22733783

[B51] Erwee M. G. Goodwin P. B. (1983). Characterisation of the *Egeria densa* Planch. leaf symplast: inhibition of the intercellular movement of fluorescent probes by group II ions. *Planta* 158 320–328. 10.1007/bf00397334 24264752

[B52] Evans M. J. Choi W.-G. Gilroy S. Morris R. (2016). A ROS-assisted calcium wave dependent on AtRBOHD and TPC1 propagates the systemic response to salt stress in *Arabidopsis* roots. *Plant Physiol.* 171 1771–1784. 10.1104/pp.16.00215 27261066 PMC4936552

[B53] Evert R. F. (1977). Phloem structure and histochemistry. *Annu. Rev. Plant Physiol. Plant Mol. Biol.* 28 199–222. 10.1146/annurev.pp.28.060177.001215

[B54] Evert R. F. (1990). “Dicotelydons,” in *Sieve Elements. Comparative Structure, Induction and Development*, eds H.-D. Behnke and R. D. Sjolund (New York, NY: Springer Verlag), 103–137.

[B55] Farrokhi N. Burton R. A. Brownfield L. Hrmova M. Wilson S. M. Bacic A. (2006). Plant cell wall biosynthesis. Genetic, biochemical and functional genomics approaches to the identification of key genes. *Plant Biotechnol. J.* 4 145–167. 10.1111/67-7652.2005.00169.x 17177793

[B56] Faulkner C. Petutschnig E. Benitez-Alfonso Y. Beck M. Robatzek S. Lipka V. (2013). LYM2-dependent chitin perception limits molecular flux via plasmodesmata. *Proc. Natl. Acad. Sci. U.S.A.* 110 9166–9170. 10.1073/pnas.1203458110 23674687 PMC3670346

[B57] Felle H. (2001). pH: signal and messenger in plant cells. *Plant Biol.* 3 577–591. 10.1055/s-2001-19372

[B58] Felle H. Bertl A. (1986). Light-induced cytoplasmic changes and their interrelation to the activity of the electrogenic pump in *Riccia fluitans*. *Biochim. Biophys. Acta* 848 176–182. 10.1016/0005-2728(86)90217-3

[B59] Fisher D. B. Frame J. M. (1984). A guide to the use of exuding-stylet technique in phloem physiology. *Planta* 161 385–393. 10.1007/bf00394567 24253836

[B60] Fisher D. B. Wu Y. Ku M. S. B. (1992). Turnover of soluble proteins in the wheat sieve tube. *Plant Physiol.* 100 1433–1441. 10.1104/pp.100.3.1433 16653142 PMC1075803

[B61] Foyer C. Walker D. Spencer C. Mann B. (1982). Observations on the phosphate status and intracellular pH of intact cells, protoplasts and chloroplasts from photosynthetic tissue using phosphorus-31 nuclear magnetic resonance. *Biochem. J.* 202 429–434. 10.1042/bj2020429 7092824 PMC1158127

[B62] Francischetti I. M. B. Mather T. N. Ribeiro J. M. C. (2003). Cloning of a salivary gland metalloprotease and characterization of gelatinase and fibrin(ogen)lytic activities in the saliva of the Lime disease vector *Ixodes scapularis*. *Biochem. Biophys. Res. Commun.* 305 869–875. 10.1016/s0006-291x(03)00857-x 12767911 PMC2903890

[B63] Froelich D. F. Mullendore D. L. Jensen K. H. Ross-Elliott T. J. Anstead J. A. Thompson G. A. (2011). Phloem ultrastructure and pressure flow: SEO proteins aggregations do not affect translocation. *Plant Cell* 23 4428–4445. 10.1105/tpc.111.093179 22198148 PMC3269875

[B64] Furch A. C. U. Hafke J. B. Schulz A. van Bel A. J. E. (2007). Ca2+-mediated remote control of reversible sieve tube occlusion in *Vicia faba*. *J. Exp. Bot.* 58 2827–2938. 10.1093/jxb/erm143 17615409

[B65] Furch A. C. U. Hafke J. B. van Bel A. J. E. (2008). Plant- and stimulus-specific variations in remote-controlled sieve-tube occlusion. *Plant Sign. Behav.* 3 858–861. 10.4161/psb.3.10.6040 PMC2634397 19704522

[B66] Furch A. C. U. van Bel A. J. E. Fricker M. D. Felle H. H. Fuchs M. Hafke J. B. (2009). Sieve element Ca2+ channels as relay stations between remote stimuli and sieve tube occlusion in *Vicia faba*. *Plant Cell* 21 2118–2132. 10.1105/tpc.108.063107 19602624 PMC2729599

[B67] Furch A. C. U. van Bel A. J. E. Will T. (2015). Aphid salivary proteins are capable of degrading sieve-tube proteins. *J. Exp. Bot.* 66 533–539. 10.1093/jxb/eru487 25540441

[B68] Furch A. C. U. Zimmermann M. R. Will T. Hafke J. B. van Bel A. J. E. (2010). Remote-controlled stop of phloem mass flow by biphasic occlusion in *Cucurbita maxima*. *J. Exp. Bot.* 61 3697–3798. 10.1093/jxb/erq181 20584788 PMC2921205

[B69] Gaudioso-Pedraza R. Benitez-Alfonso Y. (2014). A phylogenetic approach to study the origin and localization of plasmodesmata-localized glucosyl hydrolases family 17. *Front. Plant Sci.* 5:212 10.3389/fpls.214.00212 PMC4033164 24904609

[B70] Gaupels F. Buhtz A. Knauer T. Desmukh S. Waller F. van Bel A. J. E. (2008). Adaptation of aphid stylectomy for analysis of proteins and mRNAs in barley phloem sap. *J. Exp. Bot.* 59 3297–3306. 10.1093/jxb/ern181 18632729 PMC2529238

[B71] Gerhardt R. Stitt M. Heldt H. W. (1987). Subcellular metabolite levels in spinach leaves. *Plant Physiol.* 83 399–407. 10.1104/pp.83.2.399 16665257 PMC1056369

[B72] Giavalisco P. Kapitza K. Kolasa A. Buhtz A. Kehr J. (2006). Towards the proteome of *Brassica napus* phloem sap. *Proteomics* 6 896–909. 10.1002/pmic.200500155 16400686

[B73] Golecki B. Schulz A. Karstens-Behrens U. Kollmann R. (1998). Evidence for graft transmission of structural phloem proteins or their precursors in heterografts of Cucurbitaceae. *Planta* 206 630–640. 10.1007/s004250050441

[B74] Görlach A. Bertram K. Hudecova S. Krizanova O. (2015). Calcium and ROS: a mutual interplay. *Redox Biol.* 6 260–271. 10.1016/j.redox.2015.08.010 26296072 PMC4556774

[B75] Goto Y. Hattori A. Mizutani S. Tsujimoto M. (2007). Aspartic acid 221 is critical in the calcium-induced modulation of the enzymatic activity of human aminopeptidase A. *J. Biol. Chem.* 282 37074–37081. 10.1074/jbc.m707251200 17932029

[B76] Guern J. Mathieu Y. Pean M. Pasquier C. Beloeil J.-C. Lallemand J.-Y. (1986). Cytoplasmic pH regulation in *Acer pseudoplatanus* cells. I. A 31P NMR description of acid load effects. *Plant Physiol.* 82 840–845. 10.1104/pp.82.3.840 16665119 PMC1056216

[B77] Gündüz E. A. Douglas A. E. (2009). Symbiotic bacteria enable insect to utilize a nutritionally-inadequate diet. *Proc. R. Soc. Lond. B Bio. Sci.* 276 982–991. 10.1098/rspb.2008.1476 PMC2664372 19129128

[B78] Guo K. Wang W. Luo L. Chen J. Guo Y. Cui F. (2014). Characterization of an aphid-specific, cysteine-rich protein enriched in salivary glands. *Biophys. Chem.* 189 25–32. 10.1016/j.bpc.2014.03.006 24731868

[B79] Guseman J. M. Lee J. S. Bogenschutz N. L. Peterson K. M. Virata R. E. Xie B. (2010). Dysregulation of cell-to-cell connectivity and stomatal patterning by loss-of-function mutation in *Arabidopsis* chorus (glucan synthase-like 8). *Development* 137 1731–1741. 10.1242/dev.49197 20430748

[B80] Hafke J. B. Furch A. C. U. Fricker M. D. van Bel A. J. E. (2009). Forisome dispersion in *Vicia faba* is triggered by Ca2+ hotspots created by concerted action of diverse Ca2+ channels in sieve elements. *Plant Sign. Behav.* 4 968–972. 10.4161/psb.4.10.9671 PMC2801364 19826217

[B81] Hafke J. B. Furch A. C. U. Reitz M. U. van Bel A. J. E. (2007). Functional sieve tube protoplasts. *Plant Physiol.* 145 703–711. 10.1104/pp.107.105940 17885083 PMC2048790

[B82] Hafke J. B. Neff R. Hütt M.-T. Lüttge U. Thiel G. (2001). Day-to-night variations of cytoplasmic pH in a crassulacean acid metabolism plant. *Protoplasma* 216 164–170. 10.100f/bf02673868 11732184

[B83] Hafke J. B. van Amerongen J.-K. Kelling F. Furch A. C. U. Gaupels F. van Bel A. J. E. (2005). Thermodynamic battle for photosynthate acquisition between sieve tubes and adjoining parenchyma in transport phloem. *Plant Physiol.* 138 1527–1537. 10.1104/pp.104.58511 15980202 PMC1176423

[B84] Hampton R. Y. (2000). ER stress response. Getting the UPR hand on misfolded proteins. *Curr. Biol.* 10 R518–R521. 10.1016/s0960-9822(00)00583-2 10898996

[B85] Hao P. Liu C. Wang Y. Chen R. Tang M. Du B. (2008). Herbivore-induced callose deposition on the sieve plats of rice: an important mechanism for host resistance. *Plant Physiol.* 146 1810–1820. 10.1104/pp.107.111484 18245456 PMC2287352

[B86] Harmel N. Létocart E. Cherqui A. Giordanengo P. Mazzucchelli G. Guillonneau F. (2008). Identification of aphid salivary proteins: a proteomic investigation of *Myzus persicae*. *Insect Mol. Biol.* 17 165–174. 10.1111/j.1365.2583.2008.00790.x 18353105

[B87] Hattori M. Nakamura M. Komatsu S. Tsuchihara K. Tamura Y. Hasegawa T. (2012). Molecular cloning of a novel calcium-binding protein in the secreted saliva of the green rice leaf hopper *Nephotettix cinticeps*. *Insect Biochem. Mol. Biol.* 42 1–9. 10.1016/j.ibmb.2011.10.001 22019571

[B88] Hedrich E. Salvador-Retacala V. Dreyer I. (2016). Electrical wiring and long-distance plant communication. *Trends Plant Sci.* 21 376–387. 10.1016/tplants.2016.01.016 26880317

[B89] Hewer A. Becker A. van Bel A. J. E. (2011). An aphid’s Odyssey – the cortical quest for the vascular bundle. *J. Exp. Biol.* 214 3868–3879. 10.1242/jeb.060913 22031752

[B90] Hewer A. Will T. van Bel A. J. E. (2010). Plant cues for aphid navigation in vascular tissues. *J. Exp. Biol.* 213 4030–4042. 10.1242/jeb.046326 21075945

[B91] Hogenhout S. A. Van der Hoorn R. A. L. Terauchi R. Kamoun S. (2009). Emerging concepts in effector biology of plant-associated organisms. *Mol. Plant Microbe Interact.* 22 115–122. 10.1094/MPMI-22-2-0115 19132864

[B92] Holdaway-Clarke T. L. Walker N. A. Hepler P. K. Overall R. L. (2000). Physiological elevations in cytoplasmic free calcium by cold or ion injection result in transient closure of higher plant plasmodesmata. *Planta* 210 329–335. 10.1007/pl00008141 10664140

[B93] Hong Z.-L. Zhang Z.-M. Olson J. M. Verma D. P. S. (2001). A novel UDP-glucose transferase is part of the callose synthese complex and interacts with phragmoplastin at the forming cell plate. *Plant Cell* 13 769–779. 10.1105/tpc.13.4.769 11283335 PMC135533

[B94] Hothorn M. van den Ende W. Lammens W. Scheffzek K. (2010). Structural insights into the pH-controlled targeting of plant cell-wall invertase by a specific inhibitor protein. *Proc. Natl. Acad. Sci. U.S.A.* 107 17427–71432. 10.1073/pnas.1004481107 20858733 PMC2951410

[B95] Huang H.-J. Liu C.-W. Cai Y.-F. Zhang M.-Z. Bao Y.-Y. Zhang C.-X. (2015). A salivary sheath protein essential for the interaction of the brown planthopper with rice plants. *Insect Biochem. Mol. Biol.* 66 77–87. 10.1016/j.ibmb.2015.10.007 26483289

[B96] Huda K. M. K. Banu M. S. A. Tuteja R. Tuteja N. (2013). Global calcium transducer P-type ATP-ases open new avenues for agriculture by regulating stress signaling. *J. Exp. Bot.* 64 3099–3109. 10.1093/jxb/ert182 23918957

[B97] Ingvardsen C. Veierskov B. (2001). Ubiquitin- and proteasome-dependent proteolysis in plants. *Physiol. Plant.* 112 451–459. 10.1034/j.1399-3054.2001.1120401.x 11473704

[B98] Itturioz X. Rozenfeld T. Michaud A. Corvol P. Llorence-Cortes C. (2001). Study of asparagine 353 in aminopeptidase A; characterization of a novel motif (GAMEN) implicated in exopeptidase specificity of mono-zinc aminopeptidases. *Biochemistry* 40 14440–14448. 10.1021/bi11409j 11724556

[B99] Jaquiéry J. Stockel S. Nouhaud P. Mieuzet L. Mahéo F. Legeai O. F. (2012). Genome scans reveal candidate regions involved in the adaptation to host plant in the pea aphid complex. *Mol. Ecol.* 21 5251–5264. 10.1111/mec.12048 23017212

[B100] Jekat S. Ernst A. M. von Bohl A. Zielonka S. Twyman E. M. Noll G. A. (2013). P-proteins in *Arabidopsis* are heteromeric structures involved in rapid sieve tube sealing. *Front. Plant Sci.* 4:225 10.3389/pls.2013.00225 PMC3700381 23840197

[B101] Kaku H. Nishizawa Y. Ishii-Minami N. Akimoto-Tomiyama C. Dohmae N. Takio K. (2006). Plant cells recognize chitin fragments for defense signalling through a plasma-membrane receptor. *Proc. Natl. Acad. Sci. U.S.A.* 103 11086–11091. 10.1073/pnas.0508882103 16829581 PMC1636686

[B102] Kauss H. (1987). Some aspects of calcium-dependent regulation in plant metabolism. *Annu. Rev. Plant Physiol.* 38 47–71. 10.1146/annurev.pp.38.060187.000403

[B103] Kempers R. Ammerlaan A. van Bel A. J. E. (1998). Symplasmic constriction and ultrastructural features of the sieve element/companion cell complex in the transport phloem of apoplasmically and symplasmically phloem-loading species. *Plant Physiol.* 116 271–278. 10.1104/pp.116.1.271

[B104] Kim H.-M. Shin D. R. Yoo O. J. Lee H. Lee J. O. (2003). Crystal structure of *Drosophila* angiotensin I-converting enzyme bound to captopril and isinopril. *FEBS Lett.* 538 65–70. 10.1016/s0014-5793(03)00128-5 12633854

[B105] Kleinig H. (1975). Filament formation in vitro of a sieve tube protein from *Cucurbita maxima* and *Cucurbita pepo*. *Planta* 127 163–170. 10.1007/BF00388377 24430373

[B106] Knoblauch M. Noll G. Müller T. Prüfer D. Schneider-Hüther I. Scharner D. (2003). ATP-independent contractile proteins from plants. *Nat. Mater.* 2 600–604. 10.1038/nmat960 12942070

[B107] Knoblauch M. Peters W. S. Ehlers K. van Bel A. J. E. (2001). Reversible calcium-regulated stopcocks in legume sieve tubes. *Plant Cell* 13 1221–1230. 10.1105/tpc.13.5.1221 11340193 PMC135563

[B108] Knoblauch M. Stubenrauch M. van Bel A. J. E. Peters W. S. (2012). Forisome performance in artificial sieve tubes. *Plant Cell Environ.* 35 1419–1427. 10.1111/j.1365-3040.2012.02499.x 22348276

[B109] Knoblauch M. van Bel A. J. E. (1998). Sieve tubes in action. *Plant Cell* 10 35–50. 10.1105/tpc.10.1.35

[B110] Kudla J. Batistic O. Hashimoto K. (2010). Calcium signals: the lead currency of plant information processes. *Plant Cell* 22 541–563. 10.1105/tpc.109.072686 20354197 PMC2861448

[B111] Kumar R. Kumar D. Hyun T. K. Kim J.-Y. (2015). Players at plasmodesmal nano-channels. *J. Plant Biol.* 58 75–86. 10.1007/s12374-014-0541-z

[B112] Kusnierczyk A. Tran D. H. T. Winge P. Jørstad T. S. Reese J. C. Troczynska J. (2011). Testing the importance of jasmonate signalling in induction of plant defences upon cabbage aphid (*Brevicoryne brassicae*) attack. *BMC Genomics* 12:423 10.1186/1471-2164-12-423 PMC3175479 21854623

[B113] Lausten P. G. Vang S. Kristensen T. (2001). Mutational analysis of the active site of human insulin-regulated aminopeptidase. *Eur. J. Biochem.* 268 98–104. 10.1046/j.1432-1327.2001.01848.x 11121108

[B114] Lawton D. M. (1978). P-protein crystals do not disperse in uninjured sieve elements in roots of runner bean (*Phaseolus multiflorus*) fixed with glutaraldehyde. *Ann. Bot.* 42 353–361.

[B115] Lecourieux D. Ranjeva R. Pugin A. (2006). Calcium in plant defence-signalling pathways. *New Phytol.* 171 249–269. 10.1111/j.1469-8137.2006.01777x 16866934

[B116] Leszczynski B. Warchol J. Niraz S. (1985). The influence of phenolic compounds on the preference of winter wheat cultivars by cereal aphids. *Insect Sci. Appl.* 6 157–158.

[B117] Levy A. Epel B. L. (2009). “Cytology of the (1-3)-β-glucan (callose) in plasmodesmata and sieve-plate pores,” in *Chemistry, Biochemistry and Biology of (13) Beta Glucans and Related Polysaccharides*, eds Bacic A. Fincher G. B. Stone B. A. (London: Academic Press), 439–463.

[B118] Lin M.-K. Lee L. Y. Lough T. J. Phinney B. S. Lucas W. J. (2009). Analysis of the pumpkin phloem proteome provides insights into angiosperm sieve tube function. *Mol. Cell. Proteom.* 8 343–356. 10.1074/mcp.m800420.mcp200 18936055

[B119] Lohaus G. Winter H. Riens B. Heldt H. W. (1995). Further studies of phloem loading process in leaves of barley and spinach. The comparison of metabolite concentrations in the apoplastic compartment with those in the cytosolic compartment and in the sieve tubes. *Bot. Acta* 108 270–275. 10.1111/j.1438-8677.1995.tb00860.x

[B120] Louis J. Shah J. (2013). *Arabidopsis thaliana*-*Myzus persicae* interaction: shaping the understanding of plant defense against phloem-feeding insects. *Front. Plant Sci.* 4:213 10.3389/fpls.2013.00213 PMC3696735 23847627

[B121] Ma R. Reese J. C. Black W. C. Brame-Cox P. (1990). Detection of pectinesterase and polygalactorunase from salivary secretions of living greenbugs, *Schizaphis graminum* (Hemiptera: Aphididae). *J. Insect Physiol.* 36 507–512. 10.1016/0022-1910(90)90102-L

[B122] Madhusudhan V. V. Miles P. W. (1998). Mobility of salivary components as a possible reason for differences in the responses of alfalfa to the spotted alfalfa aphid and pea aphid. *Entomol. Exp. Appl.* 86 25–39. 10.1046/j.1570-7458.1998.00262.x

[B123] Maffei M. E. Mithöfer A. Boland W. (2007). Insects feeding on plants: rapid signals and responses preceding the induction of phytochemical release. *Phytochemistry* 68 2946–2959. 10.1016/j.phytochem.2007.07.016 17825328

[B124] Marchetti E. Civolani S. Leis M. Chicca M. Tjallingii W. F. Pasqualini E. (2009). Tissue location of resistance in apple to the rosy apple aphid established by electrical penetration graphs. *Bull. Insectol.* 62 203–208.

[B125] Martelli G. P. Castellano M. A. (1971). Light and electron microscopy of the intracellular inclusions of cauliflower mosaic virus. *J. Gen. Virol.* 13 133–140. 10.1099/0022-1317-13-1-133 4108668

[B126] Martin B. Collar J. L. Tjallingii W. F. Fereres A. (1997). Intracellular ingestion and salivation by aphids may cause the acquisition and inoculation of non-persistently transmitted plant viruses. *J. Gen. Virol.* 78 2701–2705. 10.1099/0022-1317-78-10-2701 9349493

[B127] Mathieu Y. Guern J. Kurkdjian A. Manigault P. Manigault J. Zielinskska T. (1989). Regulation of vacuolar pH of plant cells. I. Isolation and properties of vacuoles suitable for 31P NMR studies. *Plant Physiol.* 89 19–26. 10.1104/pp.89.1.19 16666513 PMC1055792

[B128] McAinsh M. R. Pittman J. K. (2009). Shaping the calcium signature. *New Phytol.* 181 275–294. 10.1111/j.1469-8137.2008.02682.x 19121028

[B129] McEuen A. R. Hart J. W. Sabnis D. D. (1981). Calcium-binding protein in sieve-tube exudate. *Planta* 151 531–534. 10.1007/bf00387430 24302204

[B130] McLean D. L. Kinsey M. G. (1965). Identification of electrically recorded curve patterns associated with aphid salivation and ingestion. *Nature* 205 1130–1131. 10.1038/2051130a0 5833223

[B131] Medina-Ortega K. J. Walker G. P. (2015). Faba bean forisomes can function in defense against generalist aphids. *Plant Cell Environ.* 38 1167–1177. 10.1111/pce.12470 25311512

[B132] Mehrabi S. Åhman I. Jonsson L. M. V. (2016). The constitutive expression and induction of three β-1,3-glucanases by bird cherry-oat aphid in relation to aphid resistance in 15 barley breeding lines. *Arthropod Plant Interact.* 10 101–111. 10.1007/s11829-016-9415-2

[B133] Messiaen J. Read N. D. V. van Cutsem P. Trewavas A. J. (1993). Cell wall oligogalacturonides increase cytosolic free calcium in carrot protoplasts. *J. Cell Sci.* 104 365–371.

[B134] Miles P. W. (1965). Studies on the salivary physiology of plant-bugs: the salivary secretions of aphids. *J. Insect Physiol.* 11 1261–1268. 10.1016/0022-1910(65)90119-8 5828294

[B135] Miles P. W. (1972). The saliva of hemipterans. *Adv. Insect Physiol.* 9 183–255. 10.1016/s0065-2806(08)60277-5

[B136] Miles P. W. (1999). Aphid saliva. *Biol. Rev. Camb. Philos. Soc.* 74 41–85. 10.1017/S0006323198005271

[B137] Miles P. W. Oertli J. J. (1993). The significance of antioxidants in aphid-plant interaction: the redox hypothesis. *Entomol. Exp. Appl.* 67 275–283. 10.1011/j.1570.7458.1993.tb01678.x

[B138] Miles P. W. Peng Z. (1989). Studies on the salivary physiology of plant bugs: detoxification of phytochemicals by the salivary peroxidase. *J. Insect Physiol.* 35 865–872. 10.1016/0022.1910(89)90102.9 5828294

[B139] Mittler T. E. Dadd R. H. (1964). Gustatory discrimination between liquids by the aphid Myzus persicae (Sulzer). *Entomol. Exp. Appl.* 7 315–328. 10.1111/j.1570-7458.1964.tb00733.x

[B140] Moloi M. J. van der Westhuizen A. J. (2006). The reactive oxygen species are involved in resistance responses of wheat to the Russian wheat aphid. *J. Plant Physiol.* 163 1118–1125. 10.1016/j.jplph.2005.07.014 17032617

[B141] Montero M. Brini M. Marsault R. Alvarez J. Sitia R. Pozzan T. (1995). Monitoring dynamic changes in free Ca2+ concentration in the ER of intact cells. *EMBO J.* 14 5467–5475. 8521803 10.1002/j.1460-2075.1995.tb00233.x PMC394660

[B142] Moreno A. Garzo E. Fernandez-Mata G. Kassem M. Aranda M. A. Ferreres A. (2011). Aphids secrete watery saliva into plant tissues from the onset of stylet penetration. *Entomol. Exper. Appl.* 139 145–153. 10.1111/j.1570-7458.2011.01117.x

[B143] Morgan J. K. Luzio G. A. Ammar E.-D. Hunter W. B. Hall D. G. Shatters R. G. (2013). Formation of stylet sheaths in are (in air) from eight species of phytophagous hemipterans from six families (suborders: Auchenorrhyncha and Sternorrhyncha). *PLoS ONE* 8:e62444 10.1371/journal.pone.0062444 PMC3634779 23638086

[B144] Muir S. E. Sanders D. (1996). Pharmacology of Ca2+ release from red beet microsomes suggests the presence ryadonine receptor homolog in higher plants. *FEBS Lett.* 395 39–42. 10.1016/0014-5793(96)01000-9 8849685

[B145] Müller B. Groscurth S. Menzel M. Rüping B. A. Twyman R. M. Prüfer D. (2014). Molecular and ultrastructural analysis of forisome units reveals the principles of forisome assembly. *Ann. Bot.* 113 1121–1137. 10.1093/aob/mcu036 24694827 PMC4030808

[B146] Mutti N. S. Louis J. Pappan L. K. Pappan K. Begum K. Chen M. S. (2008). A protein from the salivary glands of the pea aphid, *Acyrthosiphon pisum*, is essential in feeding on a host plant. *Proc. Nat. Acad. Sci. U.S.A.* 105 9965–9969. 10.1073/pnas.0708958105 PMC2481341 18621720

[B147] Mutti N. S. Park Y. Reese J. C. Reeck G. R. (2006). RNAi knockdown of a salivary transcript leading to lethality in the pea aphid *Acyrthosiphon pisum*. *J. Insect Sci.* 6 1–7. 10.1673/031.006.3801 PMC2990334 20233093

[B148] Nadwodnik J. Lohaus G. (2008). Subcellular concentrations of sugar alcohols and sugars in relation to phloem translocation in *Plantago major*, *Plantago maritima*, *Prunus persica*, and *Apium graveolens*. *Planta* 227 1079–1089. 10.1007/s00425-007-0682-0 18188589 PMC2270920

[B149] Naessens E. Dubreuil G. Giordanengo P. Baron O. L. Minet-Kebdani N. Keller H. (2015). A secreted MIF cytokine enables aphid feeding and represses plant immune responses. *Curr. Biol.* 25 1898–1903. 10.1016/j.cub.2015.05.047 26119751

[B150] Naqvi N. Liu K. Graham R. M. Husain A. (2005). Molecular basis of exopeptidase activity in the C-terminal domain of human angiotensin I-converting enzyme: insights into the origins of its exopeptidase activity. *J. Biol. Chem.* 280 6669–6675. 10.1074/jbc.m412638200 15615692

[B151] Nicholson R. L. Hammerschmidt R. (1992). Phenolic compounds and their role in disease resistance. *Annu. Rev. Phytopathol.* 30 369–389. 10.1146/annurev.py.s0.o90192.002101

[B152] Nicholson S. J. Hartson S. D. Puterka G. J. (2012). Proteomic analysis of secreted saliva from Russian wheat aphid (*Diuraphis noxia* Kurd.) biotypes that differ in virulence to wheat. *J. Proteomics* 75 2252–2268. 10.1016/jprot.2012.01.031 22348819

[B153] Nickel W. Rabouille C. (2009). Mechanisms of regulated unconventional protein secretion by a non-vesicular and vesicular mechanism. *Nature Rev. Mol. Cell Biol.* 10 148–155. 10.1038/nrm2617 19122676

[B154] Nishimura M. (1982). pH in vacuoles isolated from castor bean endosperm. *Plant Physiol.* 70 742–744. 10.1104/pp.70.3.742 16662567 PMC1065762

[B155] Noll G. A. Fontanellaz M. E. Rüping B. Ashoub A. van Bel A. J. E. Fischer R. (2007). Spatial and temporal regulation of the forisome gene for1 in the phloem during plant development. *Plant Mol. Biol.* 65 285–294. 10.1007/s11103-007-9217-0 17694275

[B156] Pan Y. Zhu L. Luo L. Kang L. Cui F. (2015). High expression of a unique aphid protein in the salivary glands of *Acyrthosiphon pisum*. *Physiol. Mol. Plant Pathol.* 92 175–180. 10.1016/j.pmpp.2015.04.006

[B157] Peccoud J. Ollivier A. Plantegenest M. Simon J.-C. (2009). A continuum of genetic divergence from sympatric host races to species in the pea aphid complex. *Proc. Natl. Acad. Sci. U.S.A.* 106 7495–7500. 10.1073/pnas.0811117106 19380742 PMC2678636

[B158] Pélissier H. Peters W. S. Collier R. van Bel A. J. E. Knoblauch M. (2008). GFP tagging of sieve element occlusion (SEO) proteins results in green fluorescent forisomes. *Plant Cell Physiol.* 49 1699–1710. 10.1093/pcp/pcn141 18784195 PMC2582178

[B159] Peng Z. Miles P. W. (1988). Studies on the salivary physiology of plant bugs: function of the catechol oxidase of the rose aphid. *J. Insect Physiol.* 11 1027–1033. 10.1016/0022-1910(88)90202-8 5828294

[B160] Petterson J. Tjallingii W. F. Hardie J. (2007). “Host-plant selection and feeding,” in *Aphids as Crop Pests*, eds van Emden H. Harrington R. (Wallingford, CT: CAB International), 87–113.

[B161] Pieterse C. M. J. van der Does D. Zamioudis C. Leon-Reyes A. van Wees S. C. M. (2012). Hormonal modulation of plant immunity. *Annu. Rev. Cell Dev. Biol.* 28 489–521. 10.1146/annurev-cellbio-092910-154055 22559264

[B162] Pitino M. Coleman A. D. Maffei M. E. Ridout C. J. Hogenhout S. A. (2011). Silencing of aphid genes by dsRNA feeding from plants. *PLoS ONE* 6:e25709 10.1371/journal.pone.0025709 PMC3187792 21998682

[B163] Pitino M. Hogenhout S. A. (2013). Aphid protein effectors promote aphid colonization in a plant species-specific manner. *Mol. Plant Microbe Interact.* 26 130–139. 10.1094/MPMI-07-12-0172-Fl 23035913

[B164] Plieth C. Sattelmacher B. Hansen U. P. (1997). Ca2+-H+-exchange buffers in green algae. *Protoplasma* 198 107–124. 10.1007/bf01282136

[B165] Ponsen M. B. (1972). The site of potato leafroll virus multiplication in its vector, *Myzus persicae*. *Meded. Landbouwhogeschool Wageningen* 72–16, 1–147. 10.1021/pr300041t

[B166] Powell G. Tosh C. R. Hardie J. (2006). Host plant selection by aphids: behavioural, evolutionary, and applied perspectives. *Ann. Rev. Entomol.* 51 309–330. 10.1146/annurev.ento.51.110104.151107 16332214

[B167] Prince D. C. Drurey C. Zipfel C. Hogenhout S. A. (2014). The leucine-rich repeat receptor-like kinase BRASSINOSTEROID INSENSITIVE-ASSOCIATED KINASE1 and the cytochrome P450 PHYTOALEXIN DEFECIENT T3 contribute to innate immunity to aphids in *Arabidopsis*. *Plant Physiol.* 164 2207–2219. 10.1104/pp.114.235598 24586042 PMC3982773

[B168] Pyati P. Bandaani A. R. Fitches E. Gatehouse J. A. (2011). Protein digestion in cereal aphids (*Sitobion avenae*) as a target for plant defence by endogenous proteinase inhibitors. *J. Insect Physiol.* 57 881–891. 10.1016/j.jinsphys.2011.03.024 21477592

[B169] Radford J. E. Vesk M. Overall R. L. (1998). Callose deposition at plasmodesmata. *Protoplasma* 201 30–37. 10.1007/bf01280708

[B170] Rahbé Y. Sauvion N. Febvay G. Peumans W. J. Gatehouse A. M. R. (1995). Toxicity of lectins and processing of ingested proteins in the pea aphid *Acyrthosiphon pisum*. *Entom. Exp. Appl.* 76 143–155. 10.1111/j.1570-7458.1995.tb01956.x

[B171] Ramm C. Wayadande A. Baird L. Nandakumar R. Madayiputhiya N. Amundsen K. (2015). Morphology and proteome characterization of the salivary glands of the western chinch bug (Hemiptera: Blissidae). *J. Econ. Entom.* 108 2055–2064. 10.1093/jee/tov149 26470353

[B172] Rao S. A. K. Carolan J. C. Wilkinson T. L. (2013). Proteomic profiling of ceral aphid saliva reveals both ubiquitous and adaptive secreted proteins. *PLoS ONE* 8:e57413 10.1371/journal.pone.0057413 PMC3584018 23460852

[B173] Reymond P. Calandra T. (2015). Plant immune responses: aphids strike back. *Curr. Biol.* 25 R604–R606. 10.1016/j.cub.2015.05.051 26196486

[B174] Rhodes J. D. Thain J. F. Wildon D. C. (1996). The pathway for systemic electrical signal conduction in the wounded tomato plant. *Planta* 200 50–57. 10.1007/bf00196648

[B175] Richardson P. T. Baker D. A. Ho L. C. (1982). The chemical composition of cucurbit vascular exudates. *J. Exp. Bot.* 33 1239–1247. 10.1093/jxb/33.6.1239

[B176] Rinne P. L. H. Kaikuranta P. M. van der Schoot C. (2001). The shoot apical meristem restores its symplasmic organization during chilling-induced release from dormancy. *Plant J.* 26 249–264. 10.1046/j.1365-313x.2001.01022.x 11439114

[B177] Rodriguez P. A. Bos J. I. B. (2013). Toward understanding the role of aphid effectors in plant infestation. *Mol. Plant Microbe Interact.* 26 25–30. 10.1094/MPMI-05-12-0119.Fl 23035915

[B178] Rodriguez P. A. Stam R. Warbroek T. Bos J. I. B. (2014). Mp10 and Mp42 from the aphid species *Myzus persicae* trigger plant defences in *Nicotiana benthamiana* through different activities. *Mol. Plant Microbe Interact.* 27 30–39. 10.1094/MPMI-05-13-0156-R 24006884

[B179] Rüping B. Ernst A. M. Jekat S. Nordzieke S. Reineke A. R. Müller B. (2010). Molecular and phylogenetic characterization of the sieve element occlusion gene family in Fabaceae and non-Fabaceae plants. *BMC Plant Biol.* 10:219 10.1186/1471-2229-10-219 PMC3017817 20932300

[B180] Sager E. Lee J.-Y. (2014). Plasmodesmata integrated cell signalling: insights from development and environmental signals and stresses. *J. Exp. Bot.* 65 6337–6358. 10.1093/jxb/eru365 25262225 PMC4303807

[B181] Saheed S. A. Cierlik I. Larsson K. A. E. Delp G. Bradley G. Jonsson L. M. V. (2009). Stronger induction of callose deposition in barley by Russian wheat aphid than bird cherry-oat aphid is not associated with differences in callose synthase or β-1,3-glucanase transcript abundance. *Physiol. Plant.* 135 150–161. 10.1111/j.1399-3054.2008.01180.x 19055542

[B182] Sasaki T. Hayashi H. Ishikawa H. (1991). Growth and reproduction of the symbiotic and aposymbiotic pea aphids, *Acyrthosiphon pisum* maintained on artificial diets. *J. Insect Physiol.* 37 749–756. 10.1016/0022-1910(91)90109-d

[B183] Schobert C. Baker L. Szederkenyi J. Groβmann P. Komor E. Hayashi H. (1998). Identification of immunologically related proteins in sieve-tube exudate collected from monocotyledonous and dicotyledonous plants. *Planta* 206 245–252. 10.1007/s004250050396

[B184] Schobert C. Groβmann P. Gottschalk M. Komor E. Pecsvaradi A. Zur Nieden U. (1995). Sieve-tube exudate from *Ricinus communis* L. seedlings contains ubiquitin and chaperones. *Planta* 196 205–210. 10.1007/bf00201375

[B185] Schwan S. Menzel M. Fritzsche M. Heilmann A. Spohn U. (2009). Micromechanical measurements of P-protein aggregates (forisomes) from *Vicia faba* plants. *Biophys. Chem.* 139 99–105. 10.1016/s301.4622(08)00244-5 19013001

[B186] Shalla T. A. Shepherd R. J. Petersen L. J. (1980). Comparative cytology of nine isolates of cauliflower mosaic virus. *Virology* 102 381–388. 10.1016/0042-6822(80)90105-1 18631647

[B187] Sharma A. Khan A. N. Subrahmanyam S. Raman A. Taylor G. S. Fletcher M. J. (2014). Salivary proteins of plant-feeding hemipteroids – implication in phytophagy. *Bull. Entom. Res.* 104 117–136. 10.1017/S0007485313000618 24280006

[B188] Sinha D. K. Chandran P. Timm A. E. Aguirre-Rojas L. Smith M. (2016). Virulent Diuraphis noxia aphids over-express calcium signaling proteins to overcome defenses of aphid-resistant wheat plants. *PLoS ONE* 11:e0146809 10.1371/journal.pone.0146809 PMC4729530 26815857

[B189] Sjolund R. D. Shih C. Y. (1983). Freeze-fracture analysis of phloem structure in plant tissue cultures. I. The sieve element reticulum. *J. Ultrastruct. Res.* 82 111–121. 10.1016/s0022-5320(83)90101-6 6848770

[B190] Sogawa K. (1965). Studies on the salivary glands of rice plant-hoppers. 1. Morphology and histology. *Jap. J. Appl. Entomol. Zool.* 9 275–290. 10.1303/jjaez.9.275

[B191] Spiller N. J. Kimmins F. M. Llewellyn M. (1985). Fine structure of aphid stylet pathways and its use in plant resistance studies. *Entomol. Exp. Appl.* 38 293–295. 10.1111/j.1570.7458.1985.tb03534.x

[B192] Srivastava V. K. Raikwar S. Tuteja R. Tuteja N. (2016). Ectopic expression of phloem motor protein pea forisome PsSEO-F1 enhances salinity stress tolerance in tobacco. *Plant Cell Rep.* 35 1021–1041. 10.1007/s00299-016-1935-9 26825595

[B193] Stafford-Banks C. A. Rotenberg D. Johnson B. R. Whitfield A. E. Ullmann D. E. (2014). Analysis of the salivary gland transcriptome of *Frankliniella occidentalis*. *PLoS ONE* 9:e94447 10.1371/journal.pone.0094447 PMC3988053 24736614

[B194] Stonebloom S. Burch-Smith T. Kim I. Meinke D. Mindrinos M. Zambryski P. (2009). Loss of the plant DEAD-box protein SE1 leads to defective mitochondria and increased cell-to-cell transport via plasmodesmata. *Proc. Natl. Acad. Sci. U.S.A.* 106 17229–17234. 10.1073/pnas.0909229106 19805190 PMC2761335

[B195] Takemoto H. Uefune M. Ozawa R. Arimura G. I. Takabayashi J. (2013). Previous infestation of pea aphids *Acyrthosiphon pisum* on broad bean plants resulted in the increased performance of conspecific nymphs on the plants. *J. Plant Interact.* 8 370–374. 10.1080/1749145.2013.786792

[B196] Thorpe P. Cock P. J. A. Bos J. (2016). Comparative transcriptomics and proteomics of three different aphid species identifies core and diverse effector sets. *BMC Genomics* 17:172 10.1186/s12864-016-2496-6 PMC4776380 26935069

[B197] Tilsner J. Nicolas W. Rosado A. Bayer E. M. (2016). Staying tight: plasmodesmal membrane contact sites and the control of cell-to-cell connectivity. *Annu. Rev. Plant Biol.* 67 337–364. 10.1146/annurev.arplant-043015-111840 26905652

[B198] Tjallingii W. F. (2006). Salivary secretions by aphids interacting with proteins of phloem wound responses. *J. Exp. Bot.* 57 739–745. 10.1093/jxb/erj088 16467410

[B199] Tjallingii W. F. Hogen Esch T. (1993). Fine structure of aphid stylet routes in plants in correlation with EPG signals. *Physiol. Entomol.* 18 317–328. 10.1111/j.1365-3032.1993.tb00604.x

[B200] Tucker E. B. (1988). Inositol bisphosphate and inositol trisphosphate inhibit cell-to-cell passage of carboxyfluorescein in staminal hairs of *Setcreasea purpurea*. *Planta* 174 358–363. 10.1007/bf00959521 24221517

[B201] Tucker E. B. Boss W. F. (1996). Mastoparan-induced intracellular Ca2+ fluxes may regulate cell-to-cell communication in plants. *Plant Physiol.* 111 459–467. 10.1104/pp.111.2.459 12226302 PMC157856

[B202] Turner R. B. (1971). Dietary amino acid requirements of the cotton aphid, *Aphis gossypii*: the sulfur-containing amino acids. *J. Insect Physiol.* 17 2451–2456. 10.1016/0022-1910(71)90092-8

[B203] Tuteja N. Umate P. van Bel A. J. E. (2010). Forisomes: calcium powered protein complexes with potential as ‘smart’ biomaterials. *Trends Biotechnol.* 28 102–110. 10.1016/j.tibtech.2009.11.005 20004992

[B204] Urbanska A. Tjallingii W. F. Dixon A. F. G. Leszczynski B. (1998). Phenol oxidizing enzymes in the grain aphid’s saliva. *Entom. Exp. Appl.* 86 197–203. 10.1046/j.1570-7458.1998.00281.x

[B205] van Bel A. J. E. (2005). “Sieve-pore plugging mechanisms,” in *Cell-Cell Channels*, eds Baluska F. Volkmann D. Barlow P. W. (Georgetown TX: Landes), 113–118.

[B206] van Bel A. J. E. Ehlers K. (2005). “Electrical signalling via plasmodesmata,” in *Plasmodesmata*, ed. Oparka K. J. (Oxford: Blackwell), 263–278.

[B207] van Bel A. J. E. Furch A. C. U. Hafke J. B. Knoblauch M. Patrick J. W. (2011). (Questions)n on phloem biology: 2. Mass flow, molecular hopping, distribution patterns and macromolecular signalling. *Plant Sci.* 181 325–330. 10.1016/j.plantsci.2011.05.008 21889037

[B208] van Bel A. J. E. Furch A. C. U. Will T. Buxa S. V. Musetti R. Hafke J. B. (2014). Spread the news: systemic dissemination and local impact of Ca2+ signals along the phloem pathway. *J. Exp. Bot.* 65 1761–1787. 10.1093/jxb/ert425 24482370

[B209] van Bel A. J. E. van Rijen H. V. M. (1994). Microelectrode-recorded development of the symplasmic autonomy of the sieve element/companion cell complex in the stem phloem of *Lupinus luteus* L. *Planta* 192 165–175. 10.1007/bf01089631

[B210] van der Hoorn R. (2008). Plant proteases: from phenotypes to molecular mechanisms. *Annu. Rev. Plant Biol.* 59 191–223. 10.1146/annurev.arplant.59.032607.092835 18257708

[B211] Van der Westhuizen A. J. Xian X. M. Botha A.-M. (1998). β-1,3-Glucanases in wheat and resistance to the Russian wheat aphid. *Physiol. Plant* 103 125–131. 10.1034/j.1399-3054.1998.1030115.x

[B212] van Dongen J. T. Schurr U. Pfister M. Geigenberger P. (2003). Phloem metabolism and function have to cope with low internal oxygen. *Plant Physiol.* 131 1529–1543. 10.1104/pp.102.017202 12692313 PMC166912

[B213] Vandermoten S. Harmel N. Mazzucchelli G. De Pauw E. Haubruge E. Francis F. (2014). Comparative analyses of salivary proteins from three aphid species. *Insect Mol. Biol.* 23 67–77. 10.1111/imb.12061 24382153

[B214] Vaten A. Dettmer J. Wu S. Stierhof Y. D. Miyashima S. Yadav S. T. (2011). Callose biosynthesis regulates symplastic trafficking during root development. *Dev. Cell* 21 1144–1155. 10.1016/devcel.2011.10.006 22172675

[B215] Verma D. P. S. Hong Z. I. (2001). Plant callose synthase complexes. *Plant Mol. Biol.* 47 693–701. 10.1023/a:1013679111111 11785931

[B216] Via S. (1991). Specialized host plant performance of pea aphid clones is not altered by experience. *Ecology* 72 1420–1427. 10.2037/1941114

[B217] Walker G. P. Medina-Ortega K. J. (2012). Penetration of faba bean sieve elements by pea aphid does not trigger forisome dispersal. *Entom. Exp. Appl.* 144 326–335. 10.1111/j.1570-7458.2012.01297.x

[B218] Walz C. Juenger M. Schad M. Kehr J. (2002). Evidence for the presence and activity of a complete antioxidant system in mature sieve tubes. *Plant J.* 31 189–197. 10.1046/j.1365-313x.2002.01348.x 12121448

[B219] Wan J. Basu K. Mui J. Vali H. Zheng H. Laliberté J. (2015). Ultrastructural characterization of turnip mosaic virus-induced cellular rearrangements reveals membrane-bound viral particles accumulating in vacuoles. *J. Virol.* 24 12441–12456. 10.1128/JVI.02138.15 PMC4665257 26423955

[B220] Wang W. Dai H. Zhang Y. Chandrasekar T. Luo L. Hiromasa Y. (2015a). Armet is an effector protein mediating aphid-plant interactions. *FASEB J.* 29 2032–2045. 10.1096/fj.14-266023 25678626

[B221] Wang W. Luo L. Lu H. Chen S. Cui F. (2015b). Angiotensin-converting enzymes modulate aphid-plant interactions. *Sci. Rep.* 5:8885 10.1038/srep08885 PMC4351530 25744345

[B222] War A. R. Paulraj M. G. Ahmad T. Buhroo A. A. Hussain B. Ignacimuthu S. (2012). Mechanisms of plant defense against insect herbivores. *Plant Signal. Behav.* 7 1306–1320. 10.4161/psb.21663 22895106 PMC3493419

[B223] Weigel H. J. Weis E. (1984). Determination of the proton concentration difference across the tonoplast membrane of isolated vacuoles by means of 9-aminoacridine fluorescence. *Plant Sci. Lett.* 33 163–175. 10.1016/0304-4211(84)90060-3

[B224] Wensler R. J. Filshie B. K. (1969). Gustatory sense organs in the food channel of aphids. *J. Morphol.* 129 473–492. 10.1002/mor.1002/jmor.1051290406

[B225] Will T. (2016a). “Aphid techniques,” in *Biology and Ecology of Aphids*, ed. Vilcinskas A. (New York, NY: CRC Press), 255–270.

[B226] Will T. (2016b). “Function of aphid saliva in aphid-plant interaction,” in *Biology and Ecology of Aphids*, ed. Vilcinskas A. (New York, NY: CRC Press), 221–237.

[B227] Will T. Furch A. C. U. Zimmermann M. (2013). How phloem-feeding insects face the challenge of phloem-located defenses. *Front. Plant Sci.* 4:336 10.3389/fpls.2013.00336 PMC3756233 24009620

[B228] Will T. Hewer A. van Bel A. J. E. (2008). A novel perfusion system shows that aphid feeding behaviour is altered by decrease of sieve-tube pressure. *Entom. Exp. Appl.* 127 237–245. 10.1111/j.1570-7458.2008.00687.x

[B229] Will T. Kornemann S. R. Furch A. C. U. Tjallingii W. F. van Bel A. J. E. (2009). Aphid watery saliva counteracts sieve-tube occlusion: a universal phenomenon? *J. Exp. Biol.* 212 3305–3312. 10.1242/jeb.028514 19801435

[B230] Will T. Steckbauer K. Hardt M. van Bel A. J. E. (2012). Aphid gel saliva: sheath structure, protein composition and secretory dependence on stylet-tip milieu. *PLoS ONE* 7:e46903 10.1371/journal.pone.0046903 PMC3462764 23056521

[B231] Will T. Tjallingii W. F. Thönnesen A. van Bel A. J. E. (2007). Molecular sabotage of plant defense by aphid saliva. *Proc. Natl. Acad. Sci. U.S.A.* 104 10536–10541. 10.1073/pnas.0703535104 17553961 PMC1965548

[B232] Will T. van Bel A. J. E. (2006). Physical and chemical interactions between aphids and plants. *J. Exp. Bot.* 57 729–737. 10.1093/jxb/erj089 16473888

[B233] Will T. van Bel A. J. E. (2008). Induction as well as suppression: how aphid saliva may exert opposite effects on plant defense. *Plant Sign. Behav.* 3 427–430. 10.4161/psb.3.6.5473 PMC2634323 19704587

[B234] Will T. Vilcinskas A. (2015). The structural sheath protein of aphids is required for phloem feeding. *Insect Biochem. Mol. Biol.* 57 34–40. 10.1016/s0965-1748(15)00003-x 25527379

[B235] Wille B. D. Hartmann G. L. (2008). Evaluation of artificial diets for rearing *Aphis glycines* (Hemiptera: Aphididae). *J. Econ. Entom.* 101 1228–1232. 10.1093/jee/101.4.1228 18767731

[B236] Xie B. Wang X. Zhu M. Zhang Z. Hong Z. (2011). CalS7 encodes a callose synthase responsible for callose deposition in the phloem. *Plant J.* 65 1–14. 10.1111/j.1365-313x.2010.04399.x 21175885

[B237] Yamaguchi M. (2005). Role of regucalcin in maintaining cell homeostasis and function. *Int. J. Mol. Med.* 15 371–389. 10.3892/ijmm.15.3.371 15702226

[B238] Zavaliev R. Levy A. Gera A. Epel B. L. (2013). Subcellular dynamics and role of *Arabidopsis* β-1,3-glucanases in cell-to-cell movement of tobamoviruses. *Mol. Plant Microbe Interact.* 29 1016–1030. 10.1094/mpmi-03-13-0062-R 23656331

[B239] Zavaliev R. Ueki S. Epel B. L. Citovsky V. (2011). Biology of callose (β-1,3-glucan) turnover at plasmodesmata. *Protoplasma* 248 117–130. 10.1007/s00709-010-0247-0 21116665 PMC9473521

[B240] Zhang H. M. Imtiaz M. S. Laver D. R. McCurdy D. W. Oﬄer C. E. van Helden D. F. (2015). Polarized and persistent Ca2+ plumes define loci for formation of wall ingrowth papillae in transfer cells. *J. Exp. Bot.* 66 1179–1190. 10.1031/jxb/eru460 25504137 PMC4339585

[B241] Zhu-Salzman K. Salzman R. A. Ahn J. E. Koiwa H. (2004). Transcript regulation of sorghum determinants against a phloem-feeding aphid. *Plant Physiol.* 134 420–431. 10.1104/pp.103.028324 14701914 PMC316321

[B242] Zielonka S. Ernst A. M. Hawar S. Twyman R. M. Prüfer D. Noll G. A. (2014). Characterization of five subgroups of the sieve element occlusion gene family in Glycine max reveals genes encoding non-forisome proteins, forisomes and forisome tails. *Plant Mol. Biol.* 86 51–67. 10.1007/s1103-014-0211-z 24928491

[B243] Zimmermann M. H. Ziegler H. (1975). “List of sugars and sugar alcohols in sieve-tube exudates,” in *Encyclopedia of Plant Physiology*, eds Zimmermann M. H. Milburn J. A. (Berlin: Springer), 480–503.

[B244] Zimmermann M. R. Hafke J. B. van Bel A. J. E. Furch A. C. U. (2013). Interaction of xylem and phloem during exudation and wound occlusion in *Cucurbita maxima*. *Plant Cell Environ.* 36 237–247. 10.1111/j.1365-3040.2012.02571.x 22765252

